# Assessment of the Utility of Physiologically-based Pharmacokinetic Model for prediction of Pharmacokinetics in Chinese and Japanese Populations

**DOI:** 10.7150/ijms.65040

**Published:** 2021-09-24

**Authors:** Yanke Yu, Jian Lin, Chieko Muto, Yinhua Li, Yuko Mori, Rajendar K Mittapalli, Susanna Tse, Jian Liu, Bei Kang Ge, Jing Liu

**Affiliations:** 1 Pfizer Inc, La Jolla, CA, USA.; 2 Pfizer Inc, Groton, CT, USA.; 3 Pfizer R&D Japan, Tokyo, Japan.; 4 Pfizer Investment Co., LTD, China.

**Keywords:** PBPK, Chinese, Japanese, Modeling & Simulation, Ethnic PK

## Abstract

The objective for the present analyses was to evaluate the utility of physiologically-based pharmacokinetic (PBPK) modeling for prediction of the pharmacokinetics (PK) in Chinese and Japanese populations with a panel of Pfizer internal compounds. Twelve compounds from Pfizer internal development pipeline with available Westerner PK data and available PK data in at least one of the subpopulations of Japanese and Chinese populations were identified and included in the current analysis. These selected compounds represent various elimination pathways across different therapeutic areas. The Simcyp^®^ PBPK simulator was used to develop and verify the PBPK models of individual compounds. The developed models for these compounds were verified by using the clinical PK data in Westerners. The verified PBPK models were further used to predict the PK of these compounds in Chinese and Japanese populations and the predicted PK parameters were compared with the observed PK parameters. Ten of the 12 compounds had PK data in Chinese, and all the 12 compounds had PK data in Japanese. In general, the PBPK models performed well in predicting PK in Chinese and Japanese, with 8 of 10 drugs in Chinese and 7 of 12 drugs in Japanese has AAFE values less than 1.25-fold. PBPK-guided predictions of the relative PK difference were successful for 75% and 50%, respectively, between Chinese and Western and between Japanese and Western of the tested drugs using 0.8-1.25 as criteria. In conclusion, well verified PBPK models developed using data from Westerners can be used to predict the PK in Chinese and Japanese populations.

## Introduction

It is well recognized that ethnicity may have significant impact on the pharmacokinetics (PK), pharmacodynamics (PD), efficacy and safety of a candidate drug. As a result, the International Conference on Harmonization (ICH) E5 guideline “ethnic factors in the acceptability of foreign clinical data” emphasizes the assessment of ethnic factors in the acceptability of foreign clinical data, and recommends conducting bridging studies in new regions in order to extrapolate the safety and efficacy data from the population in the foreign regions to the population in the new regions for ethnically sensitive candidate medicines 1. Due to the necessity of carrying out bridging studies in the new regions and relatively longer clinical trial starting time in countries like China, significant delays in clinical drug development and new drugs approval in the new regions such as Japan and China have been observed relative to the United States or EMA regions where the drugs were originally developed 2, 3.

Quantitative modeling and simulation have been increasingly employed during drug development to facilitate decision making and inform clinical study design and label information. Physiologically based pharmacokinetic (PBPK) modeling is one of such approaches. PBPK model integrates population specific demographic and physiological data into an anatomically and physiologically realistic framework 4. Coupled with drug specific parameters, PBPK model can be used to achieve mechanistic representation of the behavior of the drug in biological systems 5. A well verified and validated PBPK model can be extrapolated with confidence to predict PK in other unstudied scenarios, e.g., predicting the drug-drug interaction risk 6, predicting exposure in pediatrics 7, and predicting exposure in renal and hepatic impairment populations 8.

Several commercially available PBPK modeling software platforms such as Simcyp^®^ have developed population files for different races and ethnicities including White, Chinese and Japanese to enable PBPK modeling in these populations. Interethnic physiological differences including the weight and height distribution, liver volume, metabolizing enzymes and transporters abundance, allelic frequencies and phenotypes of polymorphic metabolizing enzymes and transporters, gastrointestinal transit times, and plasma protein composition were considered when building up the White/Chinese/Japanese population profiles to truly represent the characteristics of each subpopulation 9-15. For instance, different CYP2C19 or CYP2D6 poor metabolizer frequencies in East Asians (<5% for CYP2C19 and ~1% for CYP2D6) vs Whites (13-30% for CYP2C19 and 5-10% for CYP2D6) were incorporated 16. Therefore, PBPK modeling represents an attractive tool to predict PK of a drug in the unstudied ethnic populations, and thus potentially eliminate the necessity for carrying out the bridging PK study in the new regions.

The objective of the present analysis is to evaluate the PBPK model performance in predicting the PK in Japanese and Chinese using selected compounds with different clearance mechanisms from Pfizer internal development pipeline.

## Methods

### Compound selection

Twelve compounds from Pfizer internal development pipeline with available Westerner PK data and available PK data in at least one of the subpopulations of Japanese and Chinese populations were identified and included in the current analysis. A summary of the compounds, their elimination pathways, and exposure difference between Asian vs Western subjects are provided in Table 1. These selected compounds had various elimination pathways. Two of the compounds were eliminated primarily via renal excretion, one of the compounds was eliminated primarily via glucuronidation by uridine diphosphate-glucuronosyltransferases (UGTs), one of the compounds was eliminated primarily via renal excretion and UGTs, and the others were primarily eliminated via metabolism by cytochrome P450s (CYPs) enzymes. In addition, the active metabolite of Drug E (termed as E-M1) was also included in the current analysis.

### PBPK Model Development and Model Verification

The PBPK models for individual compounds were developed and verified against clinical studies conducted in Westerners. PBPK models for all compounds (except one) were developed using Simcyp® version 18 (Simcyp/Certara Ltd, Sheffield, UK). For compound M which has CYP2C19 as a major metabolic pathway, Simcyp^®^ ver19 was used for the PBPK modeling of this compound since this version has an updated and corrected CYP2C19 abundance in Japanese. The “Healthy Volunteer” population in Simcyp® ver18 or ver19 was used to simulate PK in Westerners during verification. Some of the key population specific parameters were included in the Supplemental Materials 1-5.

The PBPK model for each compound was developed using measured or predicted physicochemical properties such as pKa, LogP, protein binding, blood/plasma partition coefficient, etc., and incorporating metabolism and disposition characteristics for each compound. The absorption, distribution, metabolism and drug interaction properties were based on available *in vitro* and/or *in vivo* absorption, metabolism data. Systemic Vdss, CL, fractional absorption and bioavailability data from absolute bioavailability studies, as well as information related to metabolic and clearance pathways from human ADME studies, when available, were also incorporated. Verification of these PBPK models was based on simulation of clinical studies after single and/or multiple dose administrations and comparison of observed vs model predicted PK parameters and profiles. Contributions of specific metabolic pathways were confirmed based on verification with drug-drug interaction studies with enzyme inhibitors or inducers for the major metabolic pathways when available. In cases where the major metabolizing enzyme is polymorphic in nature, clinical PK data from subjects with different phenotypes (such as extensive metabolizer [EM] and poor metabolizer [PM]) were also used for model verification. The PBPK model parameters for the individual drugs were included in the Supplemental Materials 6-17.

### Simulation of PK in Japanese and Chinese

With the PBPK models verified against Westerner clinical PK, the model was then extrapolated and used to simulate PK of individual compounds in Chinese and Japanese with a virtual population of “Chinese Healthy Volunteers” or “Japanese” within the Simcyp^®^ platform. The clinical studies of a specific compound in Chinese or Japanese subjects can be carried out in multiple dose levels and/or under single dose or multiple dose conditions. For each individual study/dose cohort, the simulation was conducted and compared to the observed results. The number of subjects, age range, proportion of female subjects, dosage, and dosing regimen were matched to the actual clinical study design for each study/dose cohort of each compound. The trial number was fixed at 20 for each individual simulation for a specific trial of a given compound.

The model performance was evaluated at the study level by the ratios (AUCR and CmaxR) of the predicted geometric mean vs the observed geometric mean of the PK parameters (AUC and Cmax) for individual studies of each compound. In addition, the 99.998% confidence interval (CI) of the observed geometric mean of AUC and Cmax were constructed and compared to the predicted values 17-19, which has the advantage of taking into account both study sample size and the observed variance. The 99.998% geometric CIs were calculated using the following equations:



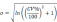





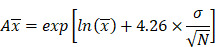





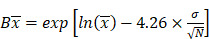



where CV% is the coefficient of variation of the observed AUC or Cmax geometric mean, σ is the standard deviation of the observed AUC or Cmax on the natural log scale, 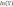 is the natural logarithm of the observed geometric mean AUC or Cmax value, N is the number of subjects in the study. The 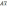 and 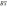 are the upper and lower boundaries of the 99.998% CI of the observed geometric mean, respectively.

Furthermore, to assess the prediction performance at compound level, the absolute average fold error (AAFE) 20 was calculated across all studies for a given drug according to the following equation:



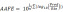



where Pred and Obs are the predicted and observed geometric mean values, respectively, of AUC and Cmax in individual studies of a specific compound.

## Results

### Model Performance in Chinese

**Figure 1:**
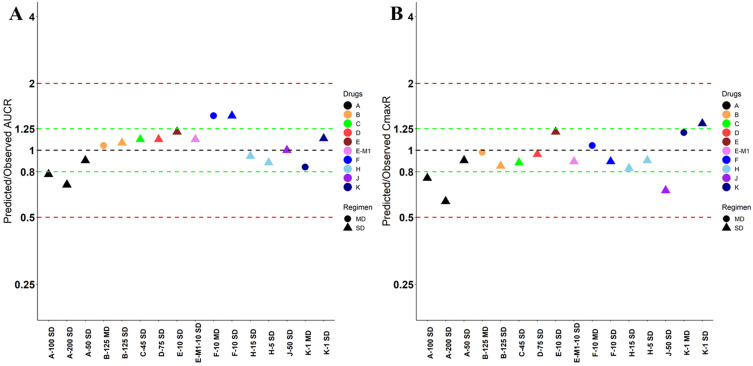
** Predicted and observed AUC ratio (A) and Cmax ratio (B) for individual dose cohorts for respective drugs in Chinese.** Dose in mg. AUCR: AUC ratio; CmaxR: Cmax ratio; SD: single dose; MD: multiple doses.

**Figure 2:**
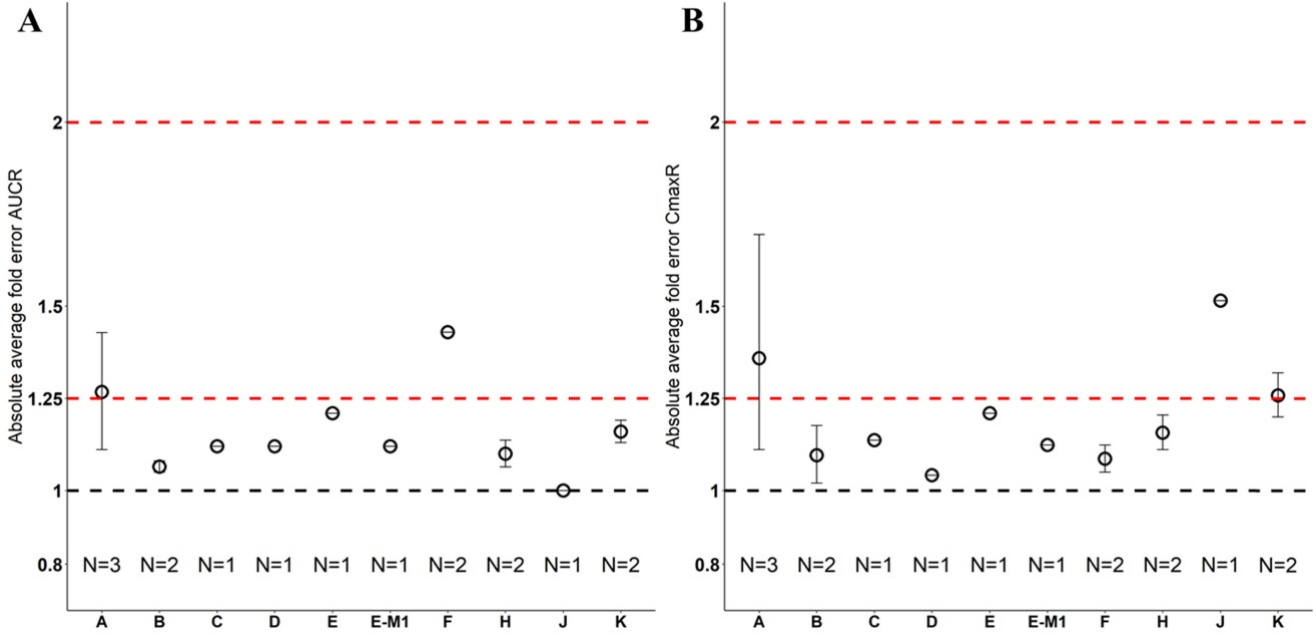
** Absolute average fold error plots for AUC ratio (A) and Cmax ratio (B) for respective drugs in Chinese.** AUCR: AUC ratio; CmaxR: Cmax ratio; N: number of cohorts.

**Figure 3:**
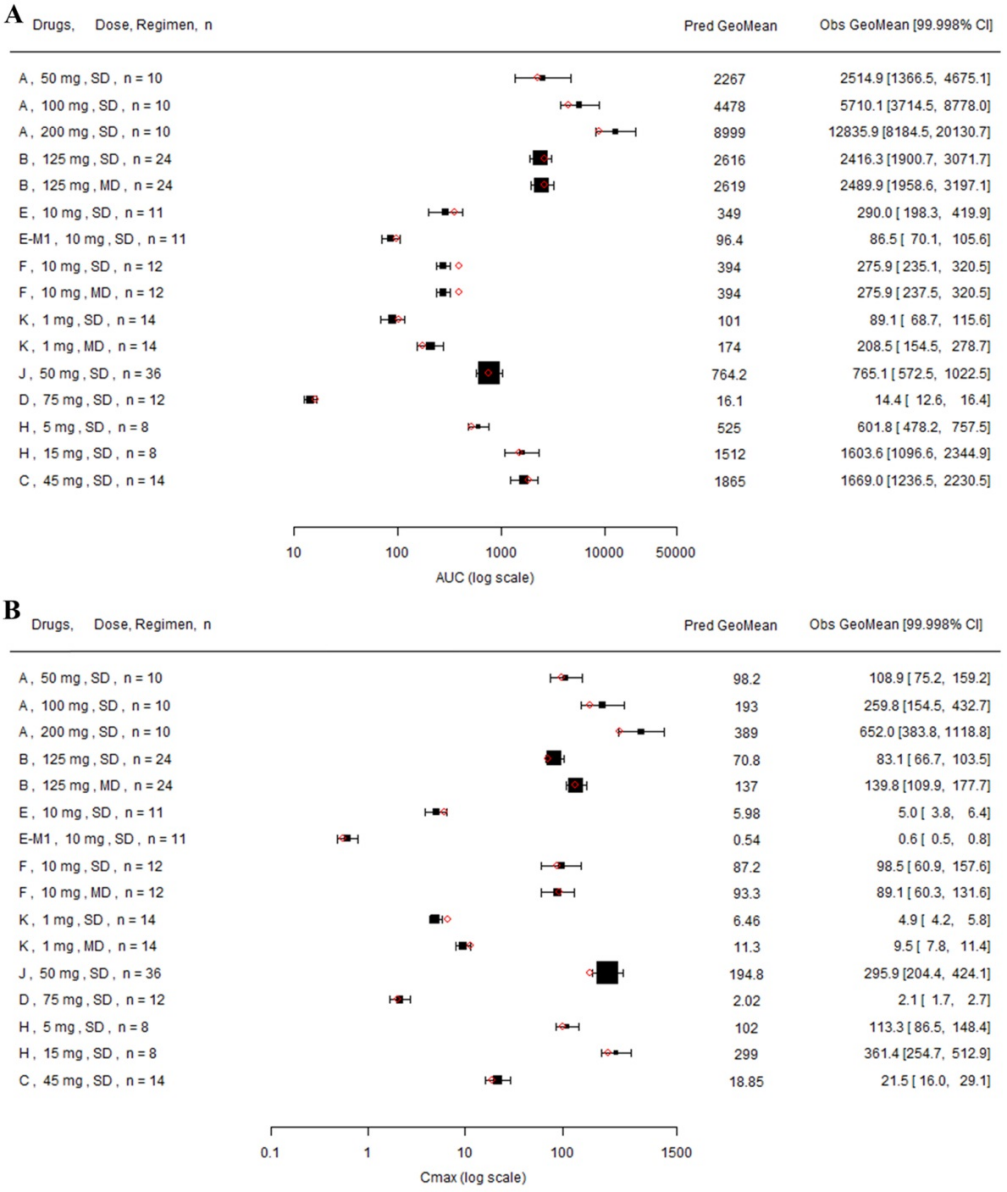
** Forest plot comparing predicted geometric mean AUC (A) and Cmax (B) vs observed geometric means and 99.998% CI in Chinese.** CI: confidence interval; GeoMean: geometric mean; n: number of subjects; Obs: observed; Pred: predicted. Red circle represents predicted geometric mean, black square represents observed geometric mean, and black error bar represent the 99.998% of observed geometric mean. The size of black square corresponds to the sample size.

Nine drugs and one metabolite of Drug E had PK in Chinese. PBPK simulation was conducted for each individual dose cohorts for the respective drugs. The predicted/observed AUC ratios (AUCR) and Cmax ratios (CmaxR) are shown in Figure 1. All of the AUC and Cmax ratios were within 0.5 to 2, with most of the values within 0.8 to 1.25. In addition, all of the drugs had AAFE values less than 1.5-fold except CmaxR of Drug E, which was 1.69-fold (Figure 2). All the compounds except Drug F had predicted geometric mean AUC fall within 99.998% CI of observed geometric mean AUC in Chinese (Figure 3A). All the compounds except Drug J had predicted geometric mean Cmax fell within 99.998% CI of observed geometric mean Cmax in Chinese (Figure 3B). Drug K had predicted geometric mean Cmax outside the 99.998% CI of observed geometric mean Cmax at 1 mg single dose, but the predicted geometric mean Cmax was within the 99.998% CI of observed geometric mean Cmax at 1 mg multiple doses. In addition, the ratios of (Predicted Cmax in Chinese/Predicted Cmax in Westerner) vs (Observed Cmax in Chinese/Observed Cmax in Westerner) ranged from 0.74 to 1.24, and the ratios of (Predicted AUC in Chinese/Predicted AUC in Westerner) vs (Observed AUC in Chinese/Observed AUC in Westerner) ranged from 0.78 to 1.48, and the ratios of (Predicted t_1/2_ in Chinese/Predicted t_1/2_ in Westerner) vs (Observed t_1/2_ in Chinese/Observed t_1/2_ in Westerner) ranged from 0.8 to 1.22 (Supplemental Materials). PBPK-guided predictions of the relative PK difference between Chinese and Western subjects were successful for 75% of the tested drugs using 0.8-1.25 as criteria. Overall, the above results demonstrated that PBPK model performed well in predicting exposure in Chinese.

### Model Performance in Japanese

**Figure 4:**
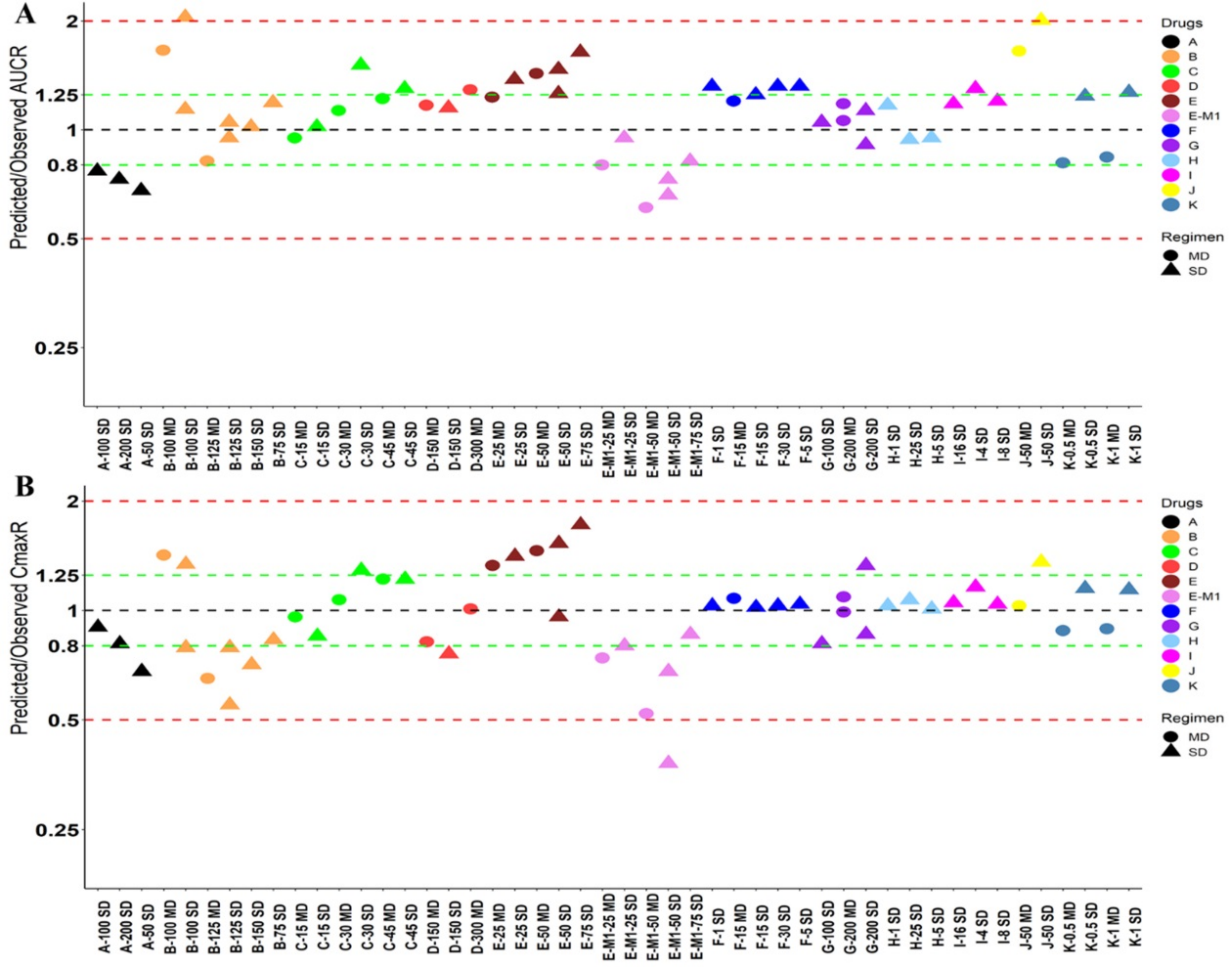
** Predicted and observed AUC ratio (A) and Cmax ratio (B) for individual dose cohorts for respective drugs in Japanese.** Dose in mg. AUCR: AUC ratio; CmaxR: Cmax ratio; SD: single dose; MD: multiple doses.

**Figure 5:**
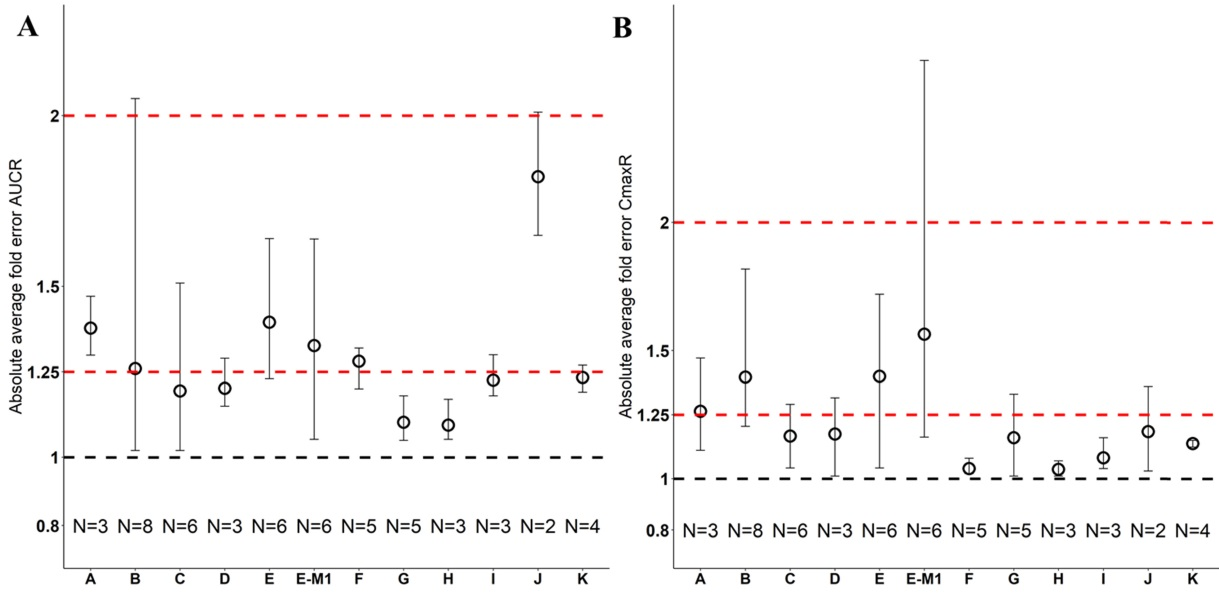
** Absolute average fold error plots for AUC ratio (A) and Cmax ratio (B) for respective drugs in Japanese.** AUCR: AUC ratio; CmaxR: Cmax ratio; N: number of cohorts.

**Figure 6:**
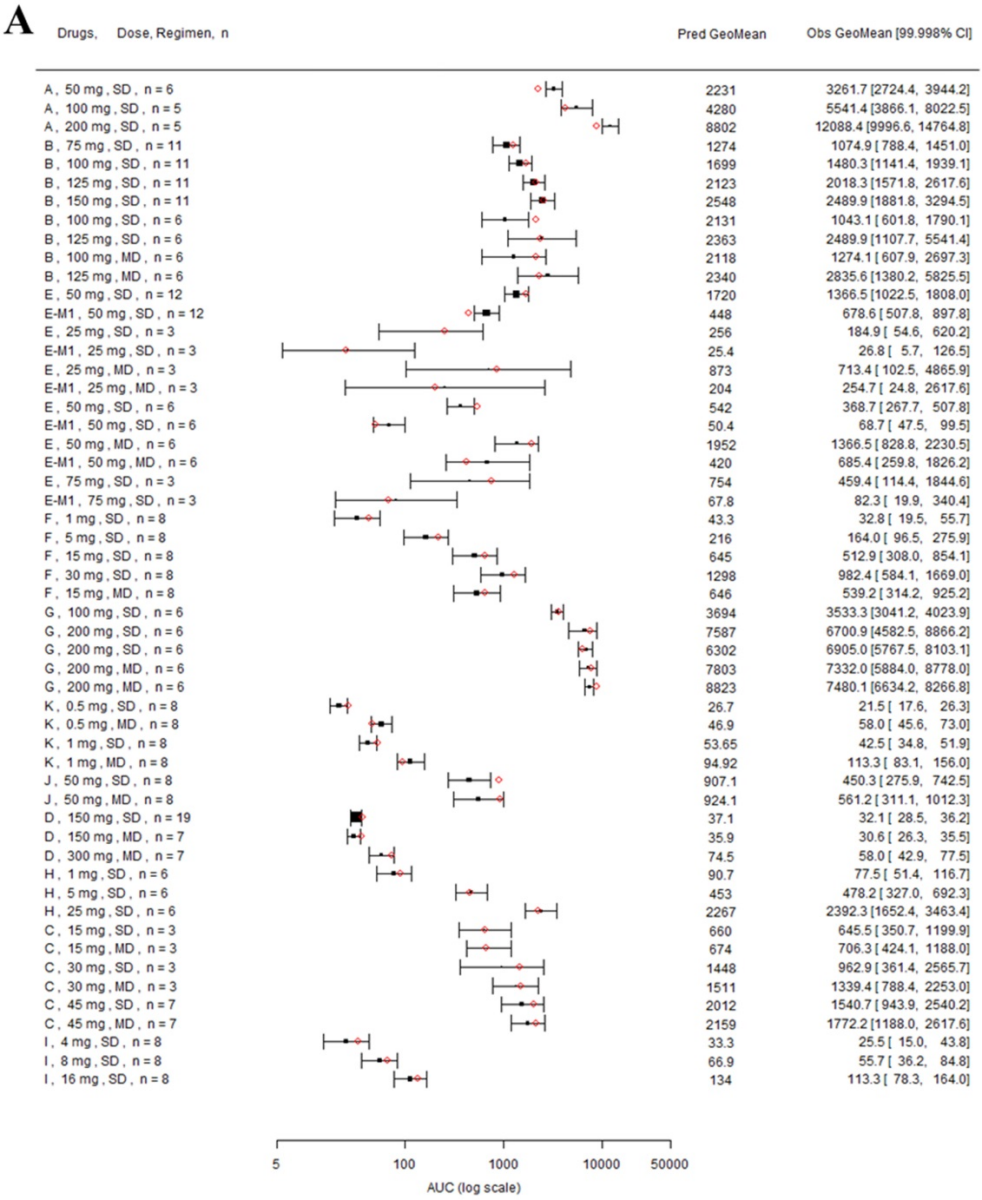

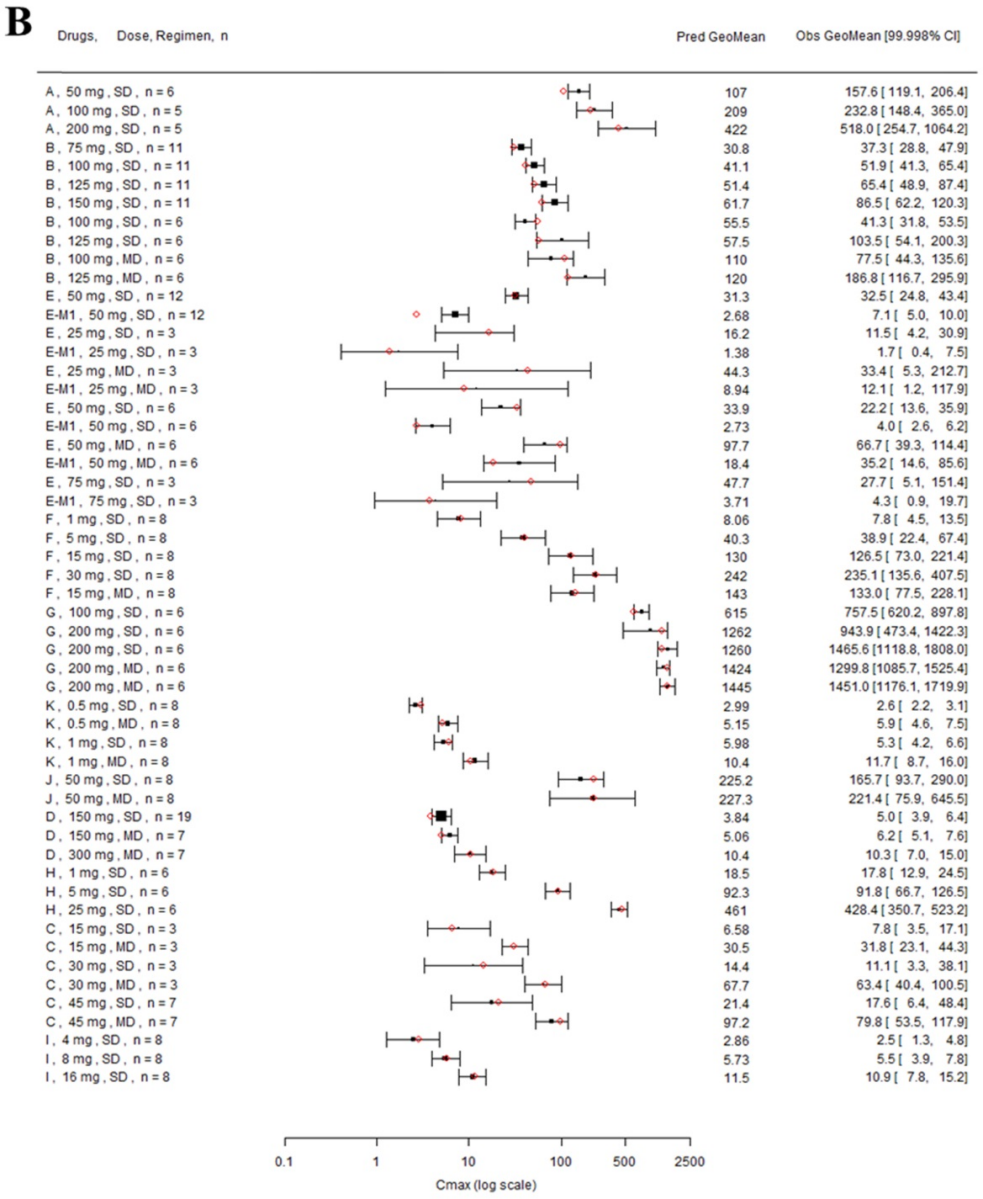
** Forest plot comparing predicted geometric mean AUC (A) and Cmax (B) vs observed geometric means and 99.998% CI in Japanese.** CI: confidence interval; GeoMean: geometric mean; n: number of subjects; Obs: observed; Pred: predicted. Red circles represent predicted geometric mean, black squares represent observed geometric mean, and black error bars represent the 99.998% of observed geometric mean. The size of black squares corresponds to the sample size.

Twelve drugs and one metabolite of Drug E had PK in Japanese. PBPK simulation was conducted for each individual dose cohorts for the respective drugs. The predicted/observed AUC ratios (AUCR) and Cmax ratios (CmaxR) are shown in Figure 4. The results of Drug M are shown separately. Most of the AUC and Cmax ratios were within 0.5 to 2, with majority of the values within 0.8 to 1.25. In addition, all of the drugs had AAFE values less than 2-fold and majority of the vales were within 1.25-fold (Figure 5). For a given drug, there was at least one dose cohort among all the tested dose cohorts having geometric mean AUC and Cmax fall within 99.998% CI of observed geometric mean AUC and Cmax, respectively, in Japanese (Figure 6). Some drugs had predicted geometric mean AUC and Cmax fall within 99.998% CI of observed geometric mean AUC and Cmax for all the dose cohorts tested in Japanese. In addition, the ratios of (Predicted Cmax in Japanese/Predicted Cmax in Westerner) vs (Observed Cmax in Japanese/Observed Cmax in Westerner) ranged from 0.76 to 1.61, the ratios of (Predicted AUC in Japanese/Predicted AUC in Westerner) vs (Observed AUC in Japanese/Observed AUC in Westerner) ranged from 0.77 to 1.76, and the ratios of (Predicted t_1/2_ in Japanese/Predicted t_1/2_ in Westerner) vs (Observed t_1/2_ in Japanese/Observed t_1/2_ in Westerner) ranged from 0.73 to 1.40 (Supplemental Materials). PBPK-guided predictions of the relative PK difference between Japanese and Western subjects were successful for 50% of the tested drugs using 0.8-1.25 as criteria. Overall, the above results demonstrated that PBPK model performed well in predicting exposure in Japanese.

### Model Performance in Chinese and Japanese with CYP Phenotypes

Drug C and M were primarily metabolized by CYP2D6 and CYP2C19, respectively. Subjects with different CYP phenotypes could have differential exposures. The CYP2D6 phenotypic information was available from the clinical PK study of Drug C in Chinese, and the CYP2C19 phenotypic information was obtained from the clinical PK study of Drug M in Japanese. The performance of the PBPK models in predicting exposure of Drug C and M in Chinese and Japanese subjects with different CYP2D6 or CYP2C19 phenotypes were examined. The predicted/observed AUCR and CmaxR were 1.21 and 0.81, respectively, for Drug C in Chinese with CYP2D6 phenotype of EM; and the predicted/observed AUCR and CmaxR were 1.15 and 0.91, respectively, for Drug C in Chinese with CYP2D6 phenotype of IM after a single oral dose of 45 mg (Table 2). After 200 mg twice daily oral dose of Drug M in Japanese with CYP2C19 phenotypes of EM, HEM, and PM (N = 5, 5 and 10, respectively), the predicted/observed AUCR were 1.35, 1.55, and 1.00, respectively, and the corresponding predicted/observed CmaxR were 0.87, 0.95, and 0.87, respectively. Due to limited sample size (N=2 for each CYP2C19 phenotypes) for Drug M in Japanese after intravenous 3 mg/kg twice daily dose, high variability was observed, and correspondingly, the predicted/observed AUCR and CmaxR showed relatively large range, ranged from 1.12 to 4.27 for AUCR and from 0.89 to 1.86 for CmaxR for the 3 CYP2C19 phenotypes in Japanese. Overall, good prediction of Drug C and M exposure in the Chinese and Japanese in the respective CYP2D6 or CYP2C19 phenotypes.

## Discussion

The contribution of PBPK modeling to facilitate drug development has been increasingly recognized. It has been implemented for better clinical study design, replacing clinical DDI studies, and assisting in regulatory decision making and informing the label languages. Previously, Matsumoto et al. demonstrated that PBPK model qualified using non-Japanese clinical data can predict PKs of nine compounds in the Japanese population, which can help with efficient trial design and Japanese Phase I studies 14. In the present analyses, the performance of PBPK models in predicting the PK in both Chinese and Japanese was evaluated. All of the 12 selected compounds with diverse elimination pathways had predicted/observed AUCR and CmaxR within 2-fold, mostly within 1.25-fold, in both Chinese and Japanese as assessed by using AAFE. In addition, 2 compounds primarily eliminated by polymorphic enzymes, CYP2D6 and CYP2C19, were assessed in different polymorphisms groups. In general, the prediction performance is reasonable for Drug C in CYP2D6 EM and IM populations. Whereas, there's varied prediction performance for drug M in CYP2C19 EM, HEM, and PM populations depending on the dosing route. Generally, there's good prediction after PO dosing which had relatively large sample size with the number of subjects of 5, 5, and 10 in CYP2C19 EM, HEM, and PM, respectively. In the CYP2C19 PM population, the prediction is the best, with predicted/observed AUCR and CmaxR as 1.00 and 0.87, respectively. Whereas, in the CYP2C19 EM/HEM populations, the prediction is less ideal, with predicted/observed AUCR ranged from 1.35 to 1.55 and CmaxR ranged from 0.87 to 0.95. In addition, after the IV dosing, the prediction performance is even worse with predicted/observed AUCR ranged from 1.12 to 4.27 and CmaxR ranged from 0.89 to 1.86 across the 3 CYP2C19 phenotypes in Japanese which could be due to limited sample size with 2 for each CYP2C19 polymorphisms. The present analyses showed that the PBPK model verified with the clinical Westerner data can be extrapolated and used to predict the PK in Chinese and Japanese.

Currently, PK bridging studies (Phase 1 PK and/or dose escalation studies or separate dose cohorts in a global Phase 1 study) are generally required in China and Japan before Chinese and Japanese can join the global pivotal studies for therapeutics initially developed in other countries, which resulted in unnecessary duplication of the clinical studies and delayed drug development and approval in these countries, possibly delaying patient access to valuable medicines. Quantitative modeling and simulation approaches may help to evaluate the necessity of conducting a bridging study in Chinese and Japanese. The PBPK model developed and verified with Westerner PK data can be used to predict the exposure in Chinese and Japanese, and assess whether any ethnic difference in PK exposure is anticipated. In addition, the popPK analysis based on the clinical data in Westerners can be used to evaluate the race effect on PK if sufficient Asian subjects were included in the study; especially, the population PK analysis can incorporate many intrinsic or extrinsic factors (e.g., CYP/transporter polymorphism, body weight, smoke status, etc.) into account to tease out confounding effect. In other words, a drug with race effect on exposure might be explained by metabolizing enzymes or transporter polymorphism differences in different ethnic populations and incorporating the CYP polymorphism differences in the popPK analysis may help elucidate that there's no significant race effect. Therefore, dose adjustment if needed should be based on CYP polymorphism but not race. For a drug with large therapeutic index, if the PBPK model predicts that exposure in Chinese and Japanese is similar to that in Westerners, and the popPK analysis confirmed that there's no significant race effect on the PK of the drug, the bridging study in Chinese and Japanese may not be needed; and Chinese and Japanese patients may directly join the global pivotal studies. In the global pivotal studies, sparse PK sampling can be collected in Chinese and Japanese to confirm the exposure in Chinese and Japanese. For a drug with narrow therapeutic index, the PBPK model predicted exposure in Chinese and Japanese as well as the popPK analysis of race effect can be used to inform more efficient study design of bridging studies, e.g., reducing sample sizes and/or the number of dose escalation cohorts if similar exposure in Chinese/Japanese was predicted compared to that in Westerners. Nevertheless, differences in the regulatory environment (e.g., different interpretation of risk-benefit balance among the countries) should also be considered when trying to use modeling and simulation approaches to streamline drug development in different countries.

The limitation of the current analyses is that all of the selected compounds had similar exposure in Asian vs Westerner or after accounting CYP enzyme polymorphisms for certain compounds. Therefore, the model performance for compounds with large exposure differences between Westerners and Asians was unknown. Nevertheless, Barter et al., demonstrated that reasonable prediction of exposure in Chinese for drugs with large exposure differences between White and Chinese 15. In addition, another limitation is that none of the selected drugs has transporter-mediated clearance. It is not clear if compounds with hepatic uptake and efflux as the major clearance pathway can be predicted equally well, which need further evaluation.

## Supplementary Material

Supplementary figures and tables.

## Figures and Tables

**Table 1:** Selected drugs in the present analysis

#	Compound	Elimination pathway	PK difference (Asian vs White)
1	A	Renal excretion, UGT conjugation, minor CYP43A4 metabolism	No difference (~-9.1% CL/F)
2	B	CYP3A metabolism; minor through SULT2A1 sulfonation	Minor difference (~-20% CL/F)
3	C	CYP2D6; minor through CYP3A4	No difference (~+8.5% CL/F)
4	D	Renal excretion	No difference
5	E	CYP3A	Minor difference (~-15.2% CL/F)
6	F	CYP3A; minor through renal excretion and CYP2C19	No difference
7	G	CYP2C9	No difference
8	H	UGT1A9 and UGTB7	No difference (~+10% AUC)
9	I	CYP2D6 and CYP3A4	No difference
10	J	CYP3A4; minor through CYP2C9	No difference
11	K	Renal excretion	No difference (~+11% AUC)
12	M	CYP2C19; minor through CYP2C9, CYP3A4	No difference (after accounting for CYP2C19 polymorphisms)

**Table 2:** Predicted and Observed AUC and Cmax of Drug C and M in Chinese and Japanese with different CYP2D6 or CYP2C19 phenotypes

Drug	Population	Dose, Route, Regimen	CYP2D6 Phenotype	N	AUC (ng*h/mL)			Cmax (ng/mL)		
					Obs	Pred	Pred/Obs Ratio	Obs	Pred	Pred/Obs Ratio
C	Chinese	45 mg, PO, SD	EM	5	1495	1809	1.21	23	18.7	0.81
			IM	8	1816	2089	1.15	21.3	19.3	0.91
			**CYP2C19 Phenotype**		AUC (µg*h/mL)			**Cmax (µg/mL)**		
M	Japanese	200 mg, PO, BID	EM	5	12.0	16.2	1.35	2.15	1.87	0.87
			HEM	5	20.0	31.0	1.55	3.36	3.18	0.95
			PM	10	65.0	65.2	1.00	6.87	5.94	0.87
		3 mg/kg, IV, BID	EM	2	5.86, 22.3	25.0	1.12, 4.27	2.36, 3.39	3.03	1.28, 0.89
			HEM	2	31.8, 26.2	52.6	1.65, 2.00	3.79, 3.93	5.45	1.44, 1.39
			PM	2	45.8, 46.8	100	2.18, 2.14	6.25, 4.94	9.19	1.47, 1.86

AUC: area under concentration-time profile; Cmax: maximum concentration; EM: extensive metabolizer; HEM: heterozygous extensive metabolizer; Obs: observed; Pred: predicted; PM: poor metabolizer. SD: single dose.
