# Supplementary material for: Assessment of the Utility of Physiologically-based Pharmacokinetic Model for prediction of Pharmacokinetics in Chinese and Japanese Populations
Source: Int J Med Sci. 2021 Sep 24;18(16):3718–27. doi: 10.7150/ijms.65040 (PMC8579302; doi:10.7150/ijms.65040)
Supplement: Supplementary file 1 — Supplementary figures and tables. [file ijmsv18p3718s1.pdf]

Supplemental Materials

Supplemental 1. Some key systems parameters for Chinese in Simcyp® version 18

CYP Phenotype

CYP enzymes	EM (Frequency)	PM (Frequency)	IM (Frequency)	UM (Frequency)
CYP2C9	0.998	0.002	0	0
CYP2C19	0.87	0.13	0	0
CYP2D6	0.597	0.003	0.39	0.01
CYP3A4	1	0	0	0

Liver Enzyme abundance (pmol/mg-protein)

CYP enzymes	EM	PM	IM	UM
CYP2C9	60	24	0	0
CYP2C19	8	0	0	0
CYP2D6	10.5	0	3.9	20.9
CYP3A4	120	0	0	0

GFR (mL/min)

Gender	Reference value 20 ~ 30 years old
Male	130
Female	120

Supplemental 2. Some key systems parameters for Japanese in Simcyp® version 18

CYP Phenotype

CYP enzymes	EM (Frequency)	PM (Frequency)	IM (Frequency)	UM (Frequency)
CYP2C9	0.992	0.008	0	0
CYP2C19	0.82	0.18	0	0
CYP2D6	0.75	0.004	0.23	0.016
CYP3A4	1	0	0	0

Liver Enzyme abundance (pmol/mg-protein)

CYP enzymes	EM	PM	IM	UM
CYP2C9	59.2	23.5	0	0
CYP2C19	4.1	0	0	0
CYP2D6	10.5	0	3.9	20.9
CYP3A4	112.2	0	0	0

GFR (mL/min)

Gender	Reference value 20 ~ 30 years old
Male	130
Female	120

CYP: cytochrome P450; EM: extensive metabolizer; GFR: glomerular filtration rate; IM: intermediate metabolizer; PM: poor metabolizer; UM: ultra metabolizer.

Supplemental 3. Some key systems parameters for Japanese in Simcyp® version 19

CYP Phenotype

CYP enzymes	EM (Frequency)	PM (Frequency)	IM (Frequency)	UM (Frequency)
CYP2C9	0.992	0.008	0	0
CYP2C19	0.817	0.172	0	0
CYP2D6	0.74	0.004	0.24	0.016
CYP3A4	1	0	0	0

Liver Enzyme abundance (pmol/mg-protein)

CYP enzymes	EM	PM	IM	UM
CYP2C9	59.2	23.5	0	0
CYP2C19	4.4	0	0	8.7
CYP2D6	10.5	0	3.3	20.9
CYP3A4	112.2	0	0	0

GFR (mL/min)

Gender	Reference value 20 ~ 30 years old
Male	130
Female	120

CYP: cytochrome P450; EM: extensive metabolizer; GFR: glomerular filtration rate; IM: intermediate metabolizer; PM: poor metabolizer; UM: ultra metabolizer.

Supplemental 4. Some key systems parameters for Healthy Volunteer in Simcyp® version 18

CYP Phenotype

CYP enzymes	EM (Frequency)	PM (Frequency)	IM (Frequency)	UM (Frequency)
CYP2C9	0.94	0.06	0	0
CYP2C19	0.976	0.024	0	0
CYP2D6	0.865	0.082	0	0.053
CYP3A4	1	0	0	0

Liver Enzyme abundance (pmol/mg-protein)

CYP enzymes	EM	PM	IM	UM
CYP2C9	73	29	0	0
CYP2C19	14	0	0	0
CYP2D6	8	0	0	16
CYP3A4	137	0	0	0

GFR (mL/min)

Gender	Reference value 20 ~ 30 years old
Male	130
Female	120

CYP: cytochrome P450; EM: extensive metabolizer; GFR: glomerular filtration rate; IM: intermediate metabolizer; PM: poor metabolizer; UM: ultra metabolizer.

Supplemental 5. Some key systems parameters for Healthy Volunteer in Simcyp® version 19

CYP Phenotype

CYP enzymes	EM (Frequency)	PM (Frequency)	IM (Frequency)	UM (Frequency)
CYP2C9	0.94	0.06	0	0
CYP2C19	0.59	0.092	0	0.318
CYP2D6	0.865	0.082	0	0.053
CYP3A4	1	0	0	0

Liver Enzyme abundance (pmol/mg-protein)

CYP enzymes	EM	PM	IM	UM
CYP2C9	73	29	0	0
CYP2C19	4.4	0	0	8.7
CYP2D6	9.4	0	0	18.8
CYP3A4	137	0	0	0

GFR (mL/min)

Gender	Reference value 20 ~ 30 years old
Male	130
Female	120

CYP: cytochrome P450; EM: extensive metabolizer; GFR: glomerular filtration rate; IM: intermediate metabolizer; PM: poor metabolizer; UM: ultra metabolizer.

Supplemental 6A. Drug A Input Parameters for SimCYP Simulation

Parameters	Value
Molecular weight (g/mol)	263.4
LogP	0.21
Compound type	Monoprotic base
pK_a	8.34
Fu_{plasma}	0.705
B/P ratio	1.1
Fa	0.95
k_a (h^{-1})	0.171
T_{lag} (h)	0.1
Fu_{gut}	1
Q_{gut} (L/h)	11.6
V_{ss} (L/kg)	3.4
CL_{int} (CYP3A4), $\mu L \cdot min^{-1} \cdot pmol^{-1}$	0.00325
CL_{int} (Other HLM), $\mu L \cdot min^{-1} \cdot mg^{-1}$	3.789
CL_r (L/h)	11.1

Supplemental 6B. Predicted and Observed PK Parameters of Drug A In Westerner, Chinese, and Japanese

**Population/Dose**		**C_{max} (ng/mL)**	**AUC_{inf} (ng*h/mL)**	**$t_{1/2}$ (h)**	**C_{trough} (ng/mL)**
**Westerner**	**Observed**	**158**	**3931**	**9.23**	**NA**
**(100 mg PO)**	**Predicted**	**157.5**	**3946**	**9.40**	**NA**
**Chinese**	**Observed**	**259.8**	**5710**	**8.71**	**NA**
**(100 mg PO)**	**Predicted**	**193.4**	**4478**	**8.49**	**NA**
**Japanese**	**Observed**	**233**	**5541**	**9.48**	**NA**

(100 mg PO)	Predicted	209	4280	7.07	NA

C_{max} and AUC_{inf} are reported as geometric mean; $t_{1/2}$ is reported as arithmetic mean.

Supplemental 6C. Ratios of Predicted and Observed PK Parameters of Drug A In Chinese and Japanese vs Westerner

	Chinese vs Westerner			Japanese vs Westerner		
	Pred_C/Pred_W	Obs_C/Obs_W	Ratio	Pred_J/Pred_W	Obs_J/Obs_W	Ratio
C_{max}	1.23	1.64	0.75	1.33	1.47	0.90
AUC_{inf}	1.13	1.45	0.78	1.08	1.41	0.77
$t_{1/2}$	0.90	0.94	0.96	0.75	1.03	0.73
C_{trough}	NA	NA	NA	NA	NA	NA

Obs_C, Obs_J and Obs_W are observed in Chinese, Japanese, and Westerner, respectively; Pred_C, Pred_J, and Pred_W are predicted in Chinese, Japanese, and Westerner, respectively; Ratio is (Pred_C/Pred_W)/(Obs_C/Obs_W) and (Pred_J/Pred_W)/(Obs_J/Obs_W), respectively.

Supplemental 6D. Predicted and Observed PK Profiles of Drug A In Westerner, Chinese, and Japanese

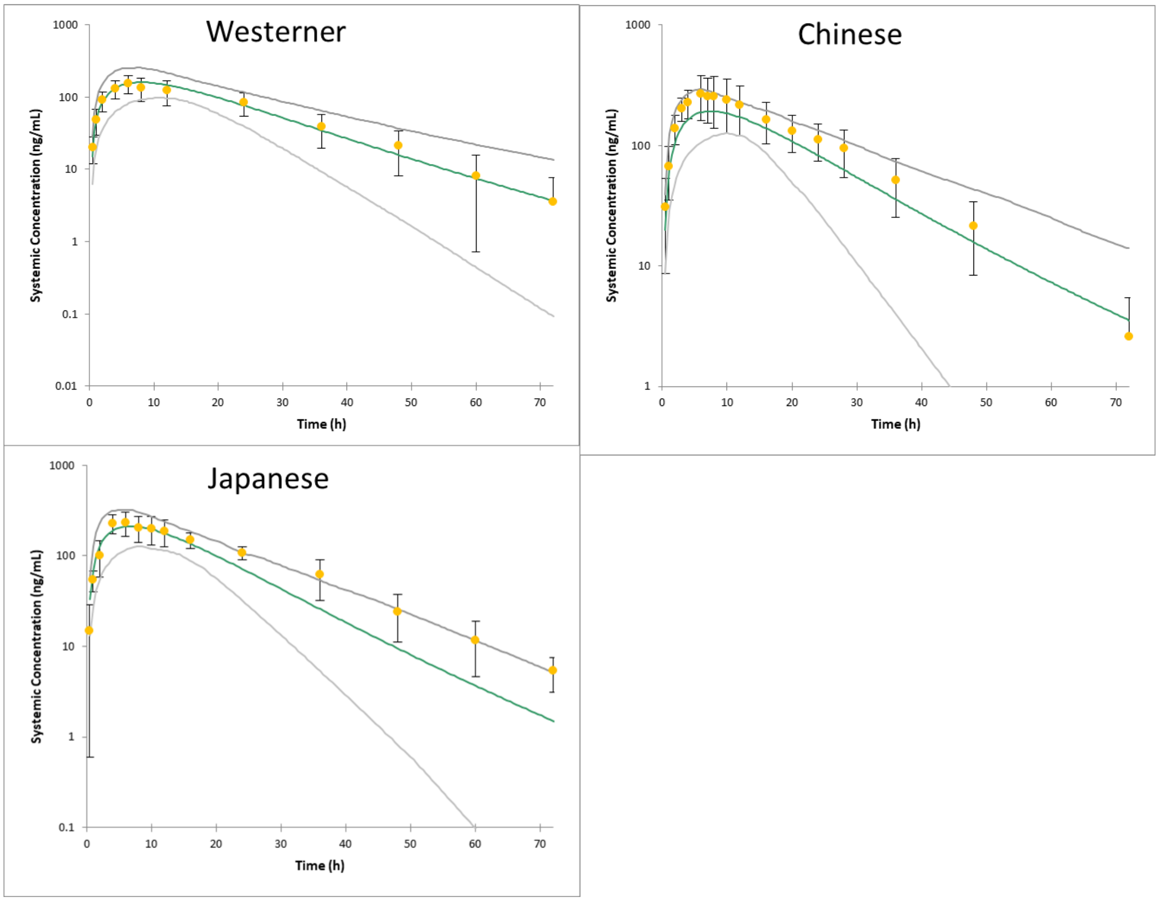

Green lines represent the predicted mean; gray lines represent the predicted 5th and 95th percentile; orange circle and error bar represent the observed mean and standard deviation.

Supplemental 7A. Drug B Input Parameters for SimCYP Simulation

Parameters	Value
Molecular weight (g/mol)	447.54

LogP	0.99
Compound type	Diprotic base
pK_a1	7.3
pK_a2	4.1
Fu_{plasma}	0.147
B/P ratio	1.63
Fa	0.85
k_a (h^{-1})	0.12
T_{lag} (h)	1.66
Fu_{gut}	0.73
Q_{gut} (L/h)	11.28
V_{ss} (L/kg)	14.3
CL_{int} (CYP3A4), $\mu L \cdot min^{-1} \cdot pmol^{-1}$	0.44
CL_{int} (Other HLM), $\mu L \cdot min^{-1} \cdot mg^{-1}$	37.65
CL_r (L/h)	6.6
CYP3A4 inhibition	--
K_{app} (μM)	10
k_{inact} (1/h)	2.16

Supplemental 7B. Predicted and Observed PK Parameters of Drug B In Westerner, Chinese, and Japanese

Population/Dose		C_{max} (ng/mL)	AUC_{inf} (ng*h/mL)	$t_{1/2}$ (h)	C_{trough} (ng/mL)
**Westerner**	**Observed**	**46.8**	**1447**	**24.54**	**64.9**
**(125 mg PO)**	**Predicted**	**47.98**	**1744**	**20.48**	**61.51**
**Chinese**	**Observed**	**83.1**	**2416**	**27.26**	**67.55**
**(125 mg PO)**	**Predicted**	**70.8**	**2616**	**22.58**	**105.77**
**Japanese**	**Observed**	**65.4**	**2018**	**23.48**	**79.5**
**(125 mg PO)**	**Predicted**	**51.4**	**2123**	**25.61**	**83.39**

C_{max}, C_{trough}, and AUC_{inf} are reported as geometric mean; $t_{1/2}$ is reported as arithmetic mean; Steady state C_{trough} is derived from multiple daily doses of Drug B at 125 mg PO.

Supplemental 7C. Ratios of Predicted and Observed PK Parameters of Drug B In Chinese and Japanese vs Westerner

	Chinese vs Westerner			Japanese vs Westerner		
	Pred_C/Pred_W	Obs_C/Obs_W	Ratio	Pred_J/Pred_W	Obs_J/Obs_W	Ratio
C_{max}	1.48	1.78	0.83	1.07	1.40	0.76
AUC_{inf}	1.5	1.67	0.90	1.22	1.39	0.88
$t_{1/2}$	1.10	1.11	0.99	1.25	0.96	1.3
C_{trough}	1.72	1.04	1.65	1.36	1.22	1.11

Obs_C, Obs_J and Obs_W are observed in Chinese, Japanese, and Westerner, respectively; Pred_C, Pred_J, and Pred_W are predicted in Chinese, Japanese, and Westerner, respectively; Ratio is (Pred_C/Pred_W)/(Obs_C/Obs_W) and (Pred_J/Pred_W)/(Obs_J/Obs_W), respectively.

Supplemental 7D. Predicted and Observed PK Profiles of Drug B In Westerner, Chinese, and Japanese

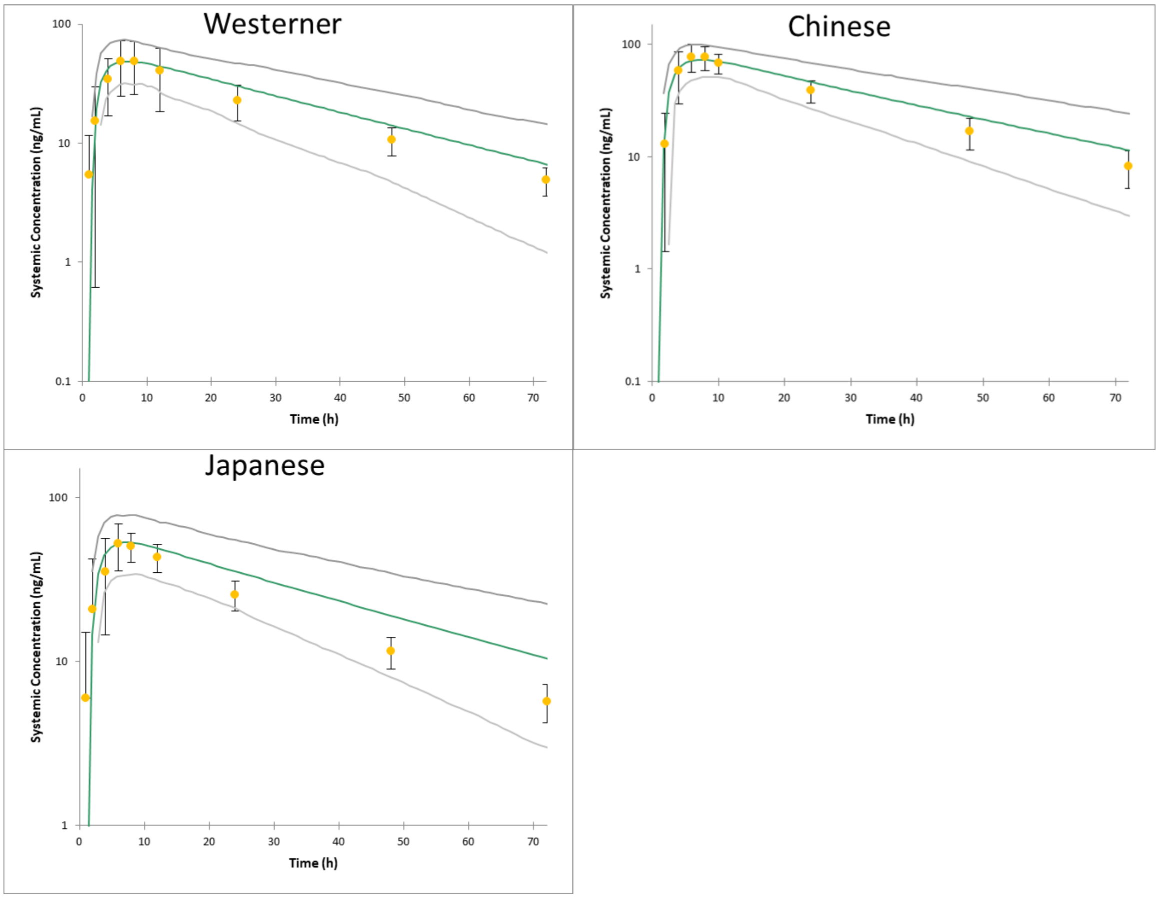

Green lines represent the predicted mean; gray lines represent the predicted 5th and 95th percentile; orange circle and error bar represent the observed mean and standard deviation.

Supplemental 7E. Predicted and Observed Ctrough of Drug B In Westerner, Chinese, and Japanese After Multiple Doses

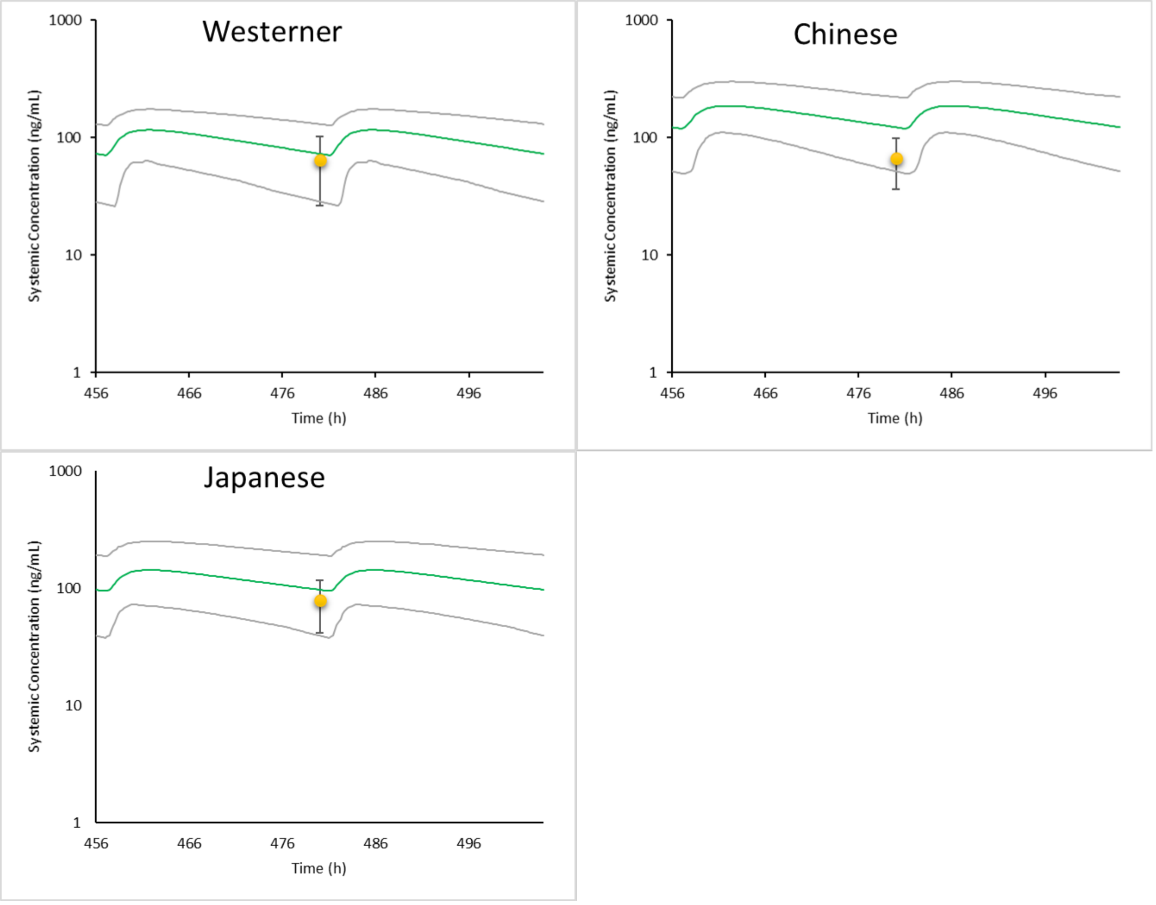

Green lines represent the predicted mean; gray lines represent the predicted 5th and 95th percentile; orange circle and error bar represent the observed mean and standard deviation.

Supplemental 8A. Drug C Input Parameters for SimCYP Simulation

Parameters	Value
Molecular weight (g/mol)	470
LogP	5.3
Compound type	Diprotic base

pK_a1	5.03
pK_a2	8.46
Fu_{plasma}	0.0192
B/P ratio	1.08
Fa	1
k_a (h^{-1})	0.2
Human Peff (x10^{-4} cm/s)	1.06
V_{ss} (L/kg)	25
V_{max} (CYP2D6), $\mu L \cdot min^{-1} \cdot mg^{-1}$	0.28
K_m (CYP2D6), μM	0.0637
CL_{int} (CYP2C9), $\mu L \cdot min^{-1} \cdot mg^{-1}$	3.5
CL_{int} (CYP3A4), $\mu L \cdot min^{-1} \cdot mg^{-1}$	23
CL_{int} (Other HLM), $\mu L \cdot min^{-1} \cdot mg^{-1}$	33
Biliary CL_{int}, $\mu L \cdot min^{-1} \cdot million^{-1}$	30.4
CL_r (L/h)	0.28
Additional system CL (L/h)	8.2
CYP3A4 Ki (μM)	8
CYP2D6 Ki (μM)	0.032

Supplemental 8B. Predicted and Observed PK Parameters of Drug C In Westerner, Chinese, and Japanese

**Population/Dose**		**C_{max} (ng/mL)**	**AUC_{inf} (ng*h/mL)**	**$t_{1/2}$ (h)**	**C_{trough} (ng/mL)**
**Westerner**	**Observed**	**17.8**	**1234**	**67.2**	**64.32**

(45 mg PO)	Predicted	16.52	1545	65.56	66.6
Chinese	Observed	21.5	1669	62.66	NA
(45 mg PO)	Predicted	18.85	1865	63.77	NA
Japanese	Observed	17.6	1541	80.03	64.9
(45 mg PO)	Predicted	21.4	2012	58.07	79.21

C_{max}, C_{trough}, and AUC_{inf} are reported as geometric mean; $t_{1/2}$ is reported as arithmetic mean; steady state C_{trough} is derived from multiple daily doses of Drug C at 45 mg PO.

Supplemental 8C. Ratios of Predicted and Observed PK Parameters of Drug C In Chinese and Japanese vs Westerner

	Chinese vs Westerner			Japanese vs Westerner		
	Pred_C/Pred_W	Obs_C/Obs_W	Ratio	Pred_J/Pred_W	Obs_J/Obs_W	Ratio
C_{max}	1.14	1.21	0.94	1.21	1.35	0.90
AUC_{inf}	1.21	1.35	0.90	1.30	1.25	1.04
$t_{1/2}$	0.97	0.93	1.04	0.89	1.19	0.75
C_{trough}	NA	NA	NA	1.19	1.01	1.18

Obs_C, Obs_J and Obs_W are observed in Chinese, Japanese, and Westerner, respectively; Pred_C, Pred_J, and Pred_W are predicted in Chinese, Japanese, and Westerner, respectively; Ratio is (Pred_C/Pred_W)/(Obs_C/Obs_W) and (Pred_J/Pred_W)/(Obs_J/Obs_W), respectively.

Supplemental 8D. Predicted and Observed PK Profiles of Drug C In Westerner, Chinese, and Japanese

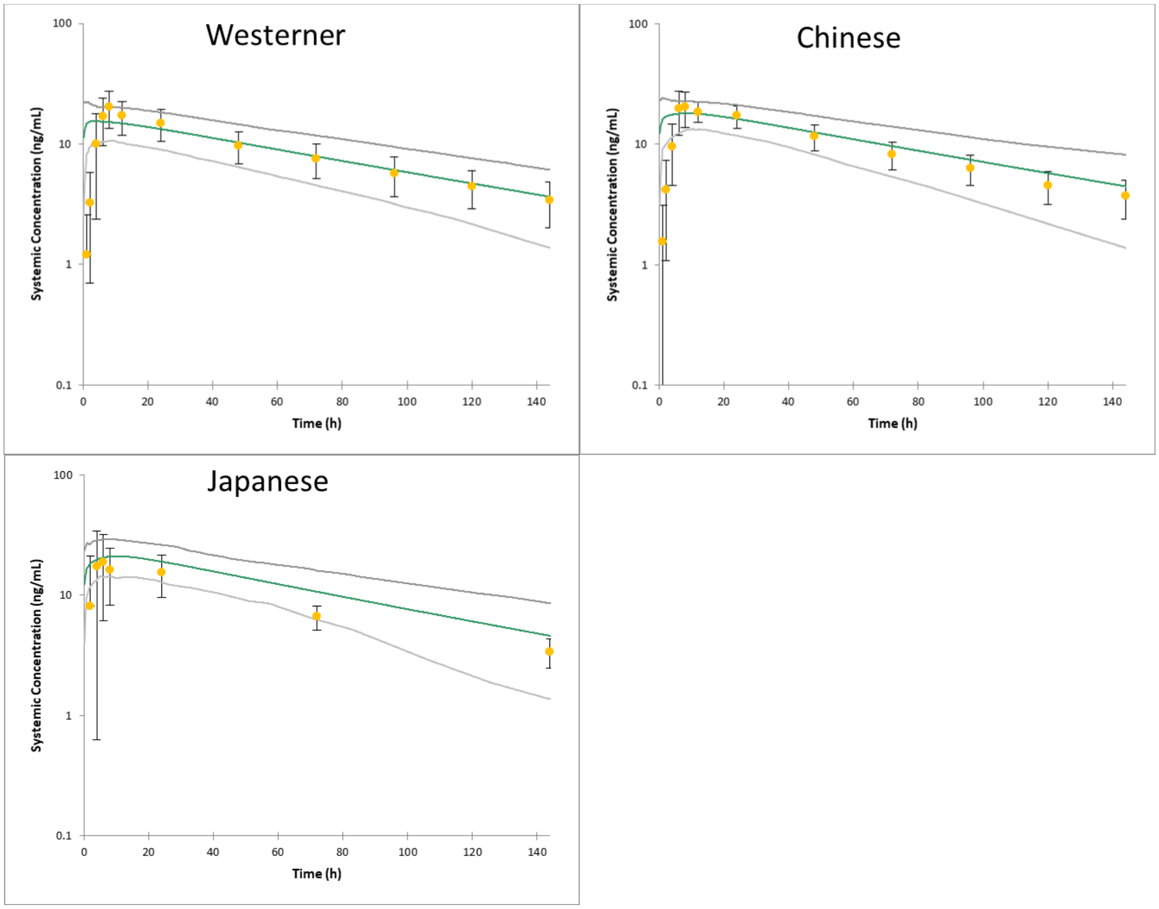

Green lines represent the predicted mean; gray lines represent the predicted 5th and 95th percentile; orange circle and error bar represent the observed mean and standard deviation.

Supplemental 8E. Predicted and Observed Ctrough of Drug C In Westerner, and Japanese After Multiple Doses

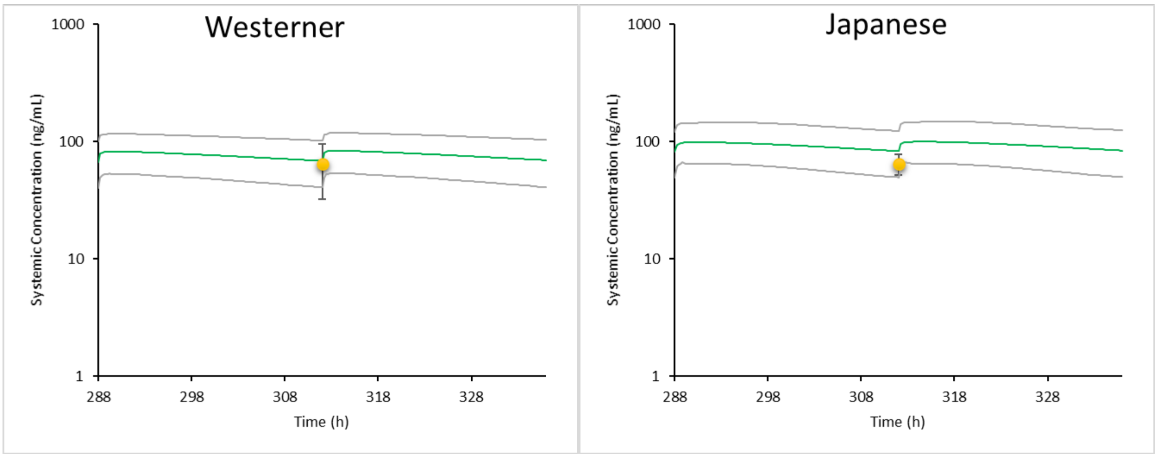

Green lines represent the predicted mean; gray lines represent the predicted 5th and 95th percentile; orange circle and error bar represent the observed mean and standard deviation.

Supplemental 9A. Drug D Input Parameters for SimCYP Simulation

Parameters	Value
Molecular weight (g/mol)	159.23
LogP	-1.35
Compound type	Ampholyte
pK_a1	4.2
pK_a2	10.6
Fu_{plasma}	1
B/P ratio	0.86
Fa	1
k_a (h^{-1})	4.18
T_{lag} (h)	0.166
Fu_{gut}	1
Q_{gut} (L/h)	10

V_{ss} (L/kg)	0.52
CL_{int} (Other HLM), $\mu L \cdot min^{-1} \cdot mg^{-1}$	0.0073
CL_r (L/h)	4.37

Supplemental 9B. Predicted and Observed PK Parameters of Drug D In Westerner, Chinese, and Japanese

**Population/Dose**		**C_{max} (ng/mL)**	**AUC_{inf} (ng*h/mL)**	**$t_{1/2}$ (h)**	**C_{trough} (ng/mL)**
**Westerner**	**Observed**	**2.95**	**21.9**	**6.10**	**1.967**
**(100 mg PO)**	**Predicted**	**2.33**	**21.74**	**5.70**	**1.63**
**Chinese**	**Observed**	**2.1**	**14.4**	**5.20**	**NA**
**(75 mg PO)**	**Predicted**	**2.02**	**16.1**	**5.12**	**NA**
**Japanese**	**Observed**	**5.0**	**32.1**	**5.82**	**1.28**
**(150 mg PO)**	**Predicted**	**3.84**	**37.1**	**6.52**	**1.39**

C_{max}, C_{trough}, and AUC_{inf} are reported as geometric mean; $t_{1/2}$ is reported as arithmetic mean; steady state C_{trough} is derived from multiple dose of Drug D at 100 mg PO three times a day in Westerner, and 150 mg PO twice a day in Japanese.

Supplemental 9C. Ratios of Predicted and Observed PK Parameters of Drug D In Chinese and Japanese vs Westerner

	Chinese vs Westerner			Japanese vs Westerner		
	Pred_C/Pred_W	Obs_C/Obs_W	Ratio	Pred_J/Pred_W	Obs_J/Obs_W	Ratio
C_{max}	0.87	0.71	1.23	1.65	1.69	0.98
AUC_{inf}	0.74	0.66	1.12	1.71	1.47	1.16
$t_{1/2}$	0.90	0.85	1.06	1.14	0.95	1.2
C_{trough}	NA	NA	NA	0.85	0.65	1.31

Obs_C, Obs_J and Obs_W are observed in Chinese, Japanese, and Westerner, respectively; Pred_C, Pred_J, and Pred_W are predicted in Chinese, Japanese, and Westerner, respectively; Ratio is (Pred_C/Pred_W)/(Obs_C/Obs_W) and (Pred_J/Pred_W)/(Obs_J/Obs_W), respectively.

Supplemental 9D. Predicted and Observed PK Profiles of Drug D In Westerner, Chinese, and Japanese

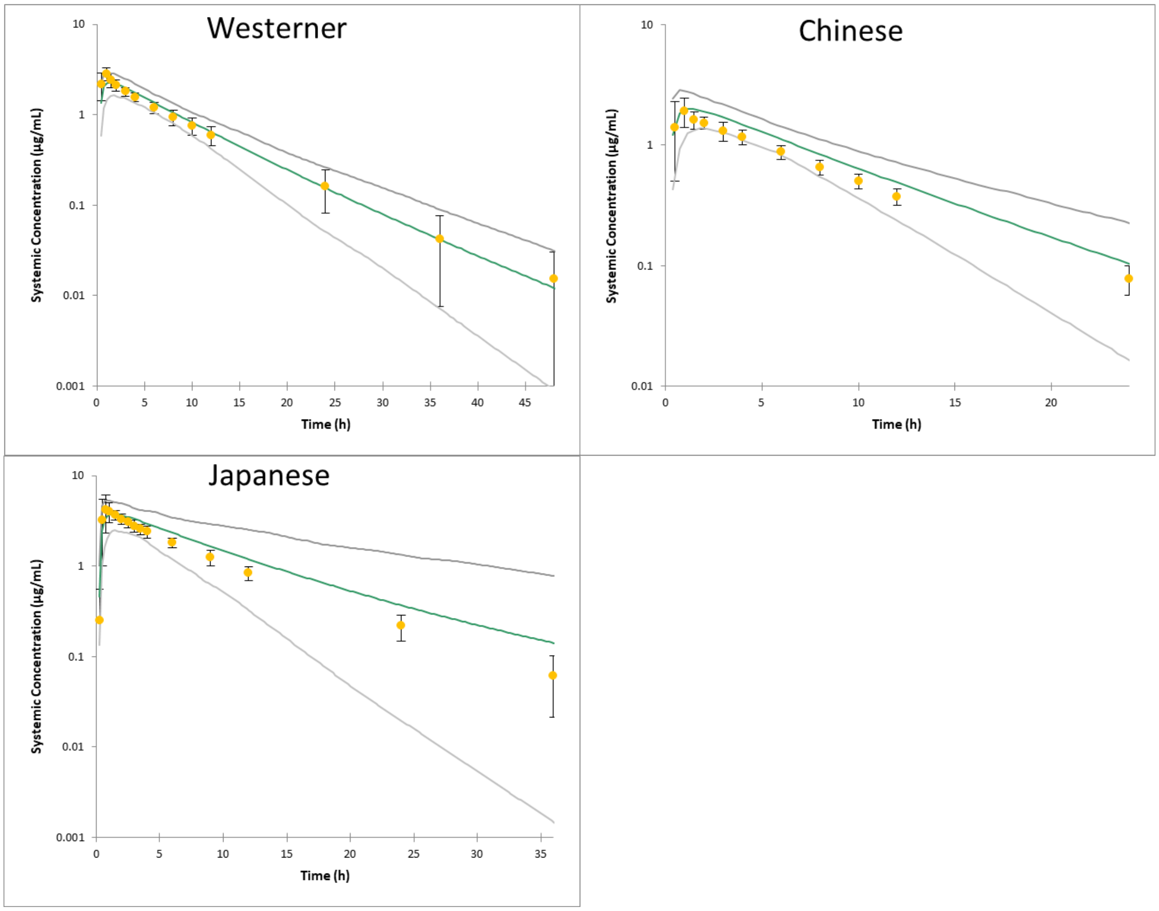

Green lines represent the predicted mean; gray lines represent the predicted 5th and 95th percentile; orange circle and error bar represent the observed mean and standard deviation.

Supplemental 9E. Predicted and Observed Ctrough of Drug D In Westerner, and Japanese After Multiple Doses

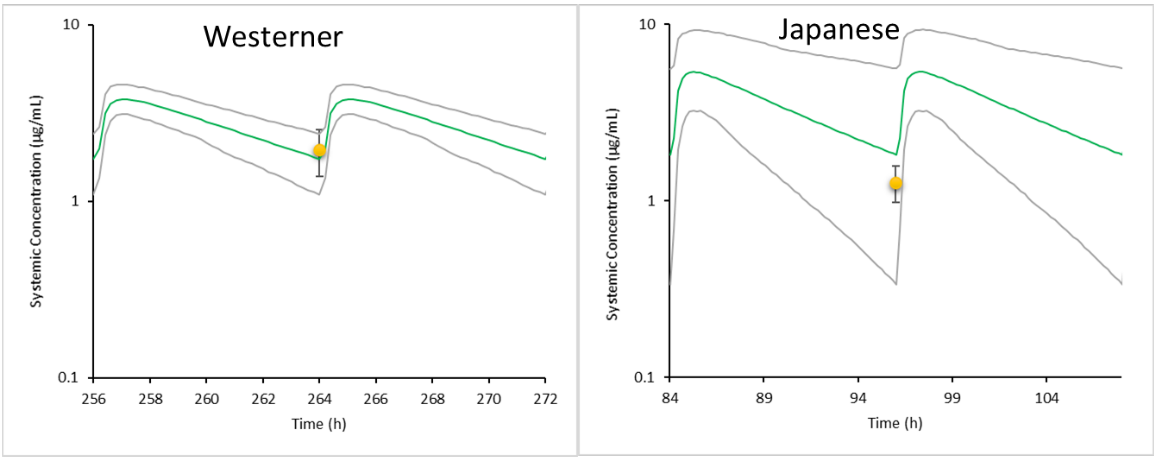

Green lines represent the predicted mean; gray lines represent the predicted 5th and 95th percentile; orange circle and error bar represent the observed mean and standard deviation.

Supplemental 10A. Drug E Input Parameters for SimCYP Simulation

Parameters	Value
Drug E	
Molecular weight (g/mol)	398.48
LogP	3.1
Compound type	Monoprotic base
pK_a	8.5
Fu_{plasma}	0.05
B/P ratio	1.17
Fa	0.791
k_a (h^{-1})	0.236
T_{lag} (h)	0.527
Fu_{gut}	0.05
Q_{gut} (L/h)	4.26

V_{ss} (L/kg)	22.9
CL_{int} (CYP3A4), $\mu L \cdot min^{-1} \cdot pmol^{-1}$	0.603
CL_{int} (Other HLM), $\mu L \cdot min^{-1} \cdot mg^{-1}$	41.6
CL_r (L/h)	4.05
Drug E-M1	
Molecular weight (g/mol)	370.42
LogP	1.88
Compound type	Monoprotic base
pK_a	10.96
Fu_{plasma}	0.1
B/P ratio	1.17
Fu_{gut}	0.1
V_{ss} (L/kg)	42.3
CL (L/h)	36.8

Supplemental 10B. Predicted and Observed PK Parameters of Drug E In Westerner, Chinese, and Japanese

**Population/Dose**		**C_{max} (ng/mL)**	**AUC_{0-24} (ng*h/mL)**	**$t_{1/2}$ (h)**	**C_{trough} (ng/mL)**
**Westerner**	**Observed**	**27.7**	**420**	**NA**	**44**
**(50 mg PO)**	**Predicted**	**29.8**	**465**	**NA**	**41.85**
**Chinese**	**Observed**	**5.0**	**290**	**NA**	**NA**
**(10 mg PO)**	**Predicted**	**5.98**	**349**	**NA**	**NA**
**Japanese**	**Observed**	**32.5**	**1367**	**NA**	**NA**
**(50 mg PO)**	**Predicted**	**31.3**	**1720**	**NA**	**NA**

AUC_{0-24} for Westerner, and AUC_{inf} for Chinese and Japanese; C_{max}, C_{trough}, and AUC are reported as geometric mean; $t_{1/2}$ is reported as arithmetic mean; steady state C_{trough} is derived from multiple dose of Drug E at 50 mg PO daily doses in Westerner.

Supplemental 10C. Ratios of Predicted and Observed PK Parameters of Drug E In Chinese and Japanese vs Westerner

	Chinese vs Westerner			Japanese vs Westerner		
	Pred_C/Pred_W	Obs_C/Obs_W	Ratio	Pred_J/Pred_W	Obs_J/Obs_W	Ratio
C_{max}	1.00	0.90	1.11	1.05	1.17	0.90
AUC_{inf}	NA	NA	NA	NA	NA	NA
$t_{1/2}$	NA	NA	NA	NA	NA	NA
C_{trough}	NA	NA	NA	NA	NA	NA

Chinese dose normalized to 50 mg for ratios calculation.

Obs_C, Obs_J and Obs_W are observed in Chinese, Japanese, and Westerner, respectively; Pred_C, Pred_J, and Pred_W are predicted in Chinese, Japanese, and Westerner, respectively; Ratio is (Pred_C/Pred_W)/(Obs_C/Obs_W) and (Pred_J/Pred_W)/(Obs_J/Obs_W), respectively.

Supplemental 10D. Predicted and Observed PK Profiles of Drug E In Westerner, Chinese, and Japanese

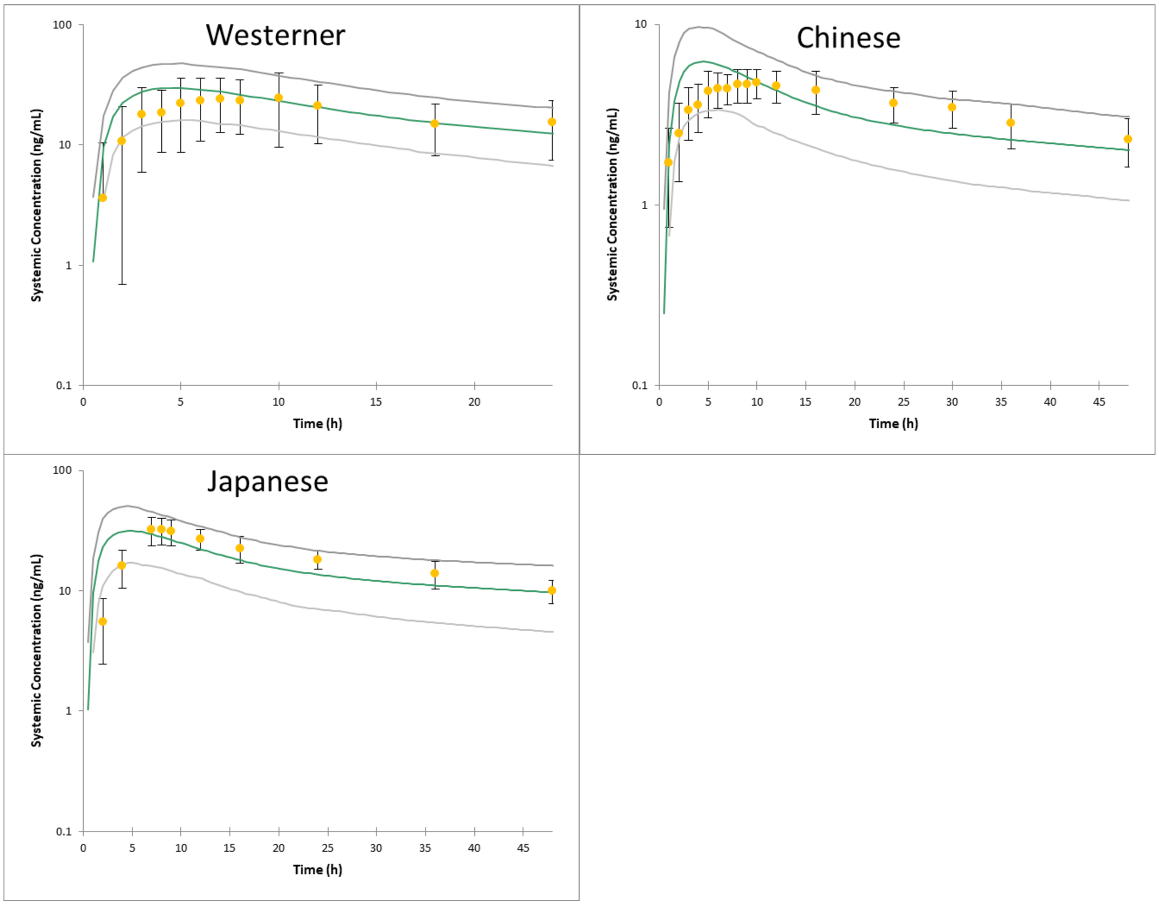

Green lines represent the predicted mean; gray lines represent the predicted 5th and 95th percentile; orange circle and error bar represent the observed mean and standard deviation.

Supplemental 10E. Predicted and Observed Ctrough of Drug E In Westerner After Multiple Doses

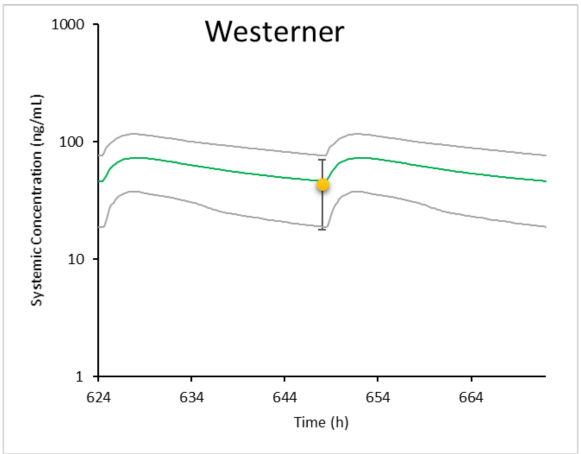

Green lines represent the predicted mean; gray lines represent the predicted 5th and 95th percentile; orange circle and error bar represent the observed mean and standard deviation.

Supplemental 11A. Drug F Input Parameters for SimCYP Simulation

Parameters	Value
Molecular weight (g/mol)	312.4
LogP	1.15
Compound type	Monoprotic base
pK_a	5.07
Fu_{plasma}	0.61
B/P ratio	1.2
Fa	0.93
k_a (h^{-1})	5.7

Fu_{gut}	1
Q_{gut} (L/h)	10
V_{ss} (L/kg)	1.24
IV CL, $L\cdot h^{-1}$	24.7
CL_{int} (CYP2C19), $\mu L\cdot min^{-1}\cdot pmol^{-1}$	0.149
CL_{int} (CYP3A4), $\mu L\cdot min^{-1}\cdot pmol^{-1}$	0.048
CL_r (L/h)	7.62

Supplemental 11B. Predicted and Observed PK Parameters of Drug F In Westerner, Chinese, and Japanese

**Population**		**C_{max} (ng/mL)**	**AUC_{inf} (ng*h/mL)**	**$t_{1/2}$ (h)**	**C_{trough} (ng/mL)**
**Westerner**	**Observed**	**88**	**289**	**2.61**	**NA**
**(10 mg PO)**	**Predicted**	**62.75**	**277.3**	**2.82**	**NA**
**Chinese**	**Observed**	**98.5**	**276**	**3.32**	**4.69**
**(10 mg PO)**	**Predicted**	**87.2**	**394**	**2.85**	**4.17**
**Japanese**	**Observed**	**127**	**513**	**3.14**	**NA**
**(15 mg PO)**	**Predicted**	**130**	**645**	**3.29**	**NA**

Cmax, C_{trough}, and AUC_{inf} are reported as geometric mean; $t_{1/2}$ is reported as arithmetic mean; steady state C_{trough} is derived from multiple dose of Drug F at 10 mg PO twice daily doses in Chinese.

Supplemental 11C. Ratios of Predicted and Observed PK Parameters of Drug F In Chinese and Japanese vs Westerner

	**Chinese vs Westerner**			**Japanese vs Westerner**		
	**Pred_C/Pred_W**	**Obs_C/Obs_W**	**Ratio**	**Pred_J/Pred_W**	**Obs_J/Obs_W**	**Ratio**
**C_{max}**	**1.39**	**1.12**	**1.24**	**1.38**	**0.96**	**1.44**
**AUC_{inf}**	**1.42**	**0.96**	**1.48**	**1.55**	**1.18**	**1.31**

**$t_{1/2}$**	**1.01**	**1.27**	**0.80**	**1.17**	**1.20**	**0.98**
**C_{trough}**	**NA**	**NA**	**NA**	**NA**	**NA**	**NA**

Japanese dose normalized to 10 mg for ratios calculation.

Obs_C, Obs_J and Obs_W are observed in Chinese, Japanese, and Westerner, respectively; Pred_C, Pred_J, and Pred_W are predicted in Chinese, Japanese, and Westerner, respectively; Ratio is (Pred_C/Pred_W)/(Obs_C/Obs_W) and (Pred_J/Pred_W)/(Obs_J/Obs_W), respectively.

Supplemental 11D. Predicted and Observed PK Profiles of Drug F In Westerner, Chinese, and Japanese

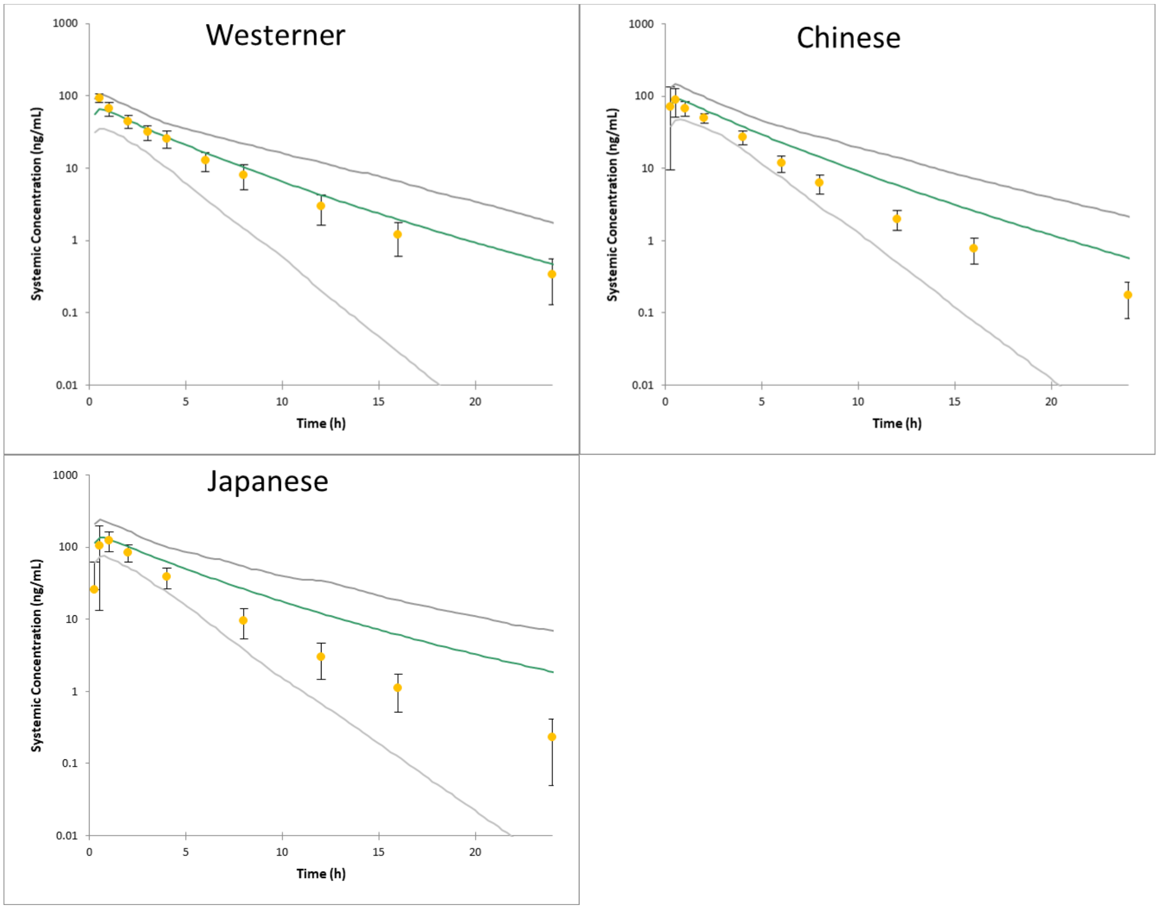

Green lines represent the predicted mean; gray lines represent the predicted 5th and 95th percentile; orange circle and error bar represent the observed mean and standard deviation.

Supplemental 11E. Predicted and Observed Ctrough of Drug F In Chinese After Multiple Doses

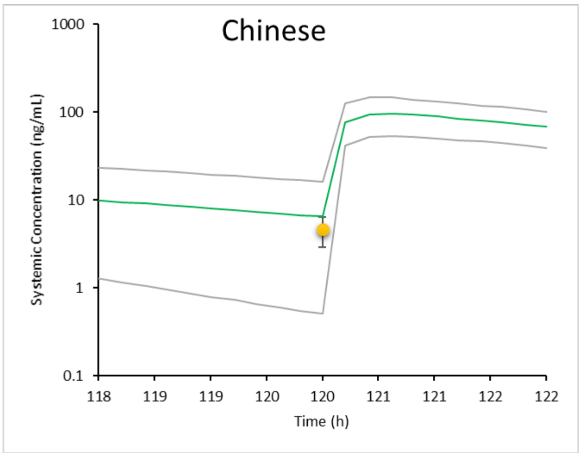

Green lines represent the predicted mean; gray lines represent the predicted 5th and 95th percentile; orange circle and error bar represent the observed mean and standard deviation.

Supplemental 12A. Drug G Input Parameters for SimCYP Simulation

**Parameters**	**Value**
Molecular weight (g/mol)	381.37
LogP	3.83
Compound type	Monoprotic acid
pK_a	10.9
Fu_{plasma}	0.027
B/P ratio	0.945
Fa	0.85
Human Peff ($x10^{-4}$ cm/s)	2.86
Fu_{gut}	1
Q_{gut} (L/h)	12.11
V_{ss} (L/kg)	5.05
V_{max} (CYP3A4), $\mu L \cdot min^{-1} \cdot pmol^{-1}$	3.08
K_m (CYP3A4), μM	16.76

CL_{int} (Other HLM), $\mu L\cdot min^{-1}\cdot mg^{-1}$	16.2
CL_r (L/h)	0.584

Supplemental 12B. Predicted and Observed PK Parameters of Drug G In Westerner, and Japanese

**Population/Dose**		**C_{max} (ng/mL)**	**AUC_{inf} (ng*h/mL)**	**$t_{1/2}$ (h)**	**C_{trough} (ng/mL)**
**Westerner**	**Observed**	**797**	**6272**	**7.57**	**NA**
**(200 mg PO)**	**Predicted**	**665.8**	**5500**	**10.9**	**NA**
**Japanese**	**Observed**	**944**	**6701**	**5.6**	**311.2**
**(200 mg PO)**	**Predicted**	**1262**	**7587**	**8.17**	**238.1**

Cmax, C_{trough}, and AUC_{inf} are reported as geometric mean; $t_{1/2}$ is reported as arithmetic mean; steady state C_{trough} is derived from multiple doses of Drug G at 200 mg PO twice daily doses in Japanese.

Supplemental 12C. Ratios of Predicted and Observed PK Parameters of Drug G In Japanese vs Westerner

Japanese vs Westerner

	Pred_J/Pred_W	Obs_J/Obs_W	Ratio
**C_{max}**	**1.90**	**1.18**	**1.61**
**AUC_{inf}**	**1.38**	**1.07**	**1.29**
**$t_{1/2}$**	**0.75**	**0.74**	**1.01**
**C_{trough}**	**NA**	**NA**	**NA**

Obs_J and Obs_W are observed in Japanese and Westerner, respectively; Pred_J and Pred_W are predicted in Japanese and Westerner, respectively; Ratio is (Pred_J/Pred_W)/(Obs_J/Obs_W).

Supplemental 12D. Predicted and Observed PK Profiles of Drug G In Westerner, Chinese, and Japanese

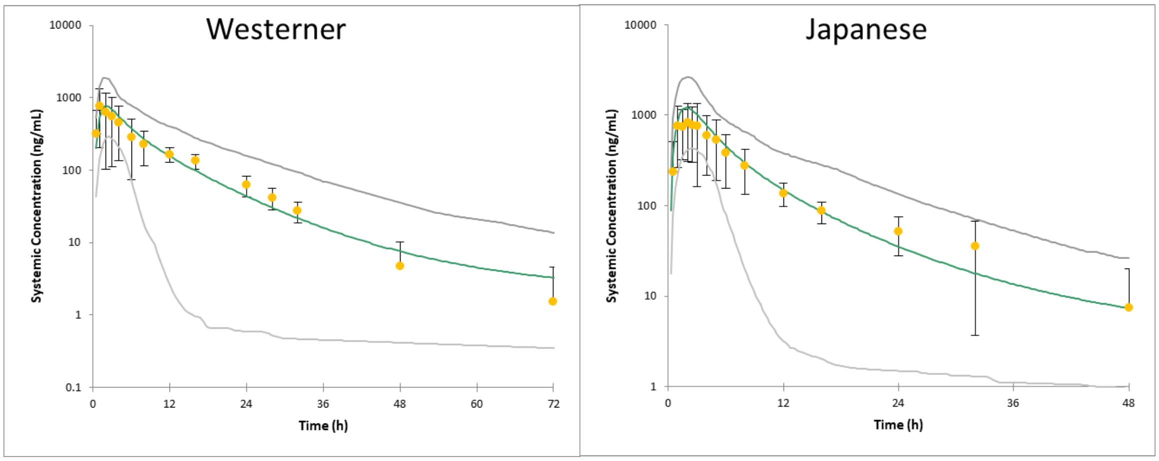

Green lines represent the predicted mean; gray lines represent the predicted 5th and 95th percentile; orange circle and error bar represent the observed mean and standard deviation.

Supplemental 12E. Predicted and Observed Ctrough of Drug G In Japanese After Multiple Doses

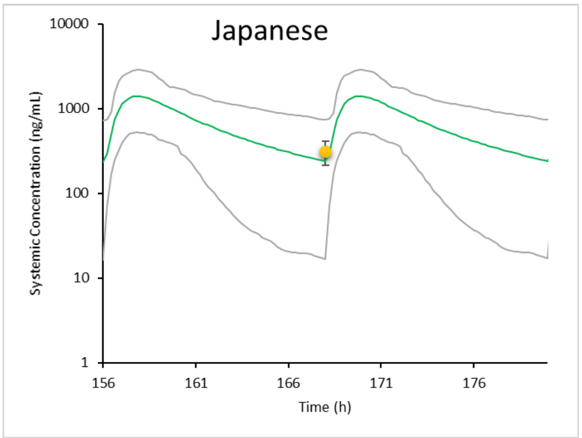

Green lines represent the predicted mean; gray lines represent the predicted 5th and 95th percentile; orange circle and error bar represent the observed mean and standard deviation.

Supplemental 13A. Drug H Input Parameters for SimCYP Simulation

**Parameters**	**Value**
Molecular weight (g/mol)	436
LogP	2.5
Compound type	Neutral
Fu_{plasma}	0.064
B/P ratio	0.66
Fa	1
k_a (h^{-1})	1.2
T_{lag} (h)	0.5
Fu_{gut}	0.064
Q_{gut} (L/h)	5.62
V_{ss} (L/kg)	1.23

CL_{iv}, L/h	11.2
CL_{int} (CYP3A4), $\mu L \cdot min^{-1} \cdot pmol^{-1}$	0.041
CL_{int} (CYP3A5), $\mu L \cdot min^{-1} \cdot pmol^{-1}$	0.006
CL_{int} (CYP2C8), $\mu L \cdot min^{-1} \cdot pmol^{-1}$	0.011
CL_{int} (UGT1A9), $\mu L \cdot min^{-1} \cdot mg^{-1}$	35
CL_{int} (UGT2B7), $\mu L \cdot min^{-1} \cdot mg^{-1}$	8
CL_r (L/h)	0.1

Supplemental 13B. Predicted and Observed PK Parameters of Drug H In Westerner, Chinese, and Japanese

**Population/Dose**		**C_{max} (ng/mL)**	**AUC_{0-24} (ng*h/mL)**	**$t_{1/2}$ (h)**	**C_{trough} (ng/mL)**
**Westerner**	**Observed**	**81.3**	**398**	**NA**	**NA**
**(5 mg PO)**	**Predicted**	**79.3**	**382.4**	**NA**	**NA**
**Chinese**	**Observed**	**113**	**602**	**NA**	**NA**
**(5 mg PO)**	**Predicted**	**102**	**525**	**NA**	**NA**
**Japanese**	**Observed**	**91.8**	**478**	**NA**	**NA**
**(5 mg PO)**	**Predicted**	**92.3**	**453**	**NA**	**NA**

C_{max} and AUC_{inf} are reported as geometric mean; $t_{1/2}$ is reported as arithmetic mean

Supplemental 13C. Ratios of Predicted and Observed PK Parameters of Drug H In Chinese and Japanese vs Westerner

	Chinese vs Westerner			Japanese vs Westerner		
	Pred_C/Pred_W	Obs_C/Obs_W	Ratio	Pred_J/Pred_W	Obs_J/Obs_W	Ratio
C_{max}	1.29	1.39	0.93	1.16	1.13	1.03
AUC_{inf}	1.37	1.51	0.91	1.18	1.20	0.98
$t_{1/2}$	NA	NA	NA	NA	NA	NA
C_{trough}	NA	NA	NA	NA	NA	NA

Obs_C, Obs_J and Obs_W are observed in Chinese, Japanese, and Westerner, respectively; Pred_C, Pred_J, and Pred_W are predicted in Chinese, Japanese, and Westerner, respectively; Ratio is (Pred_C/Pred_W)/(Obs_C/Obs_W) and (Pred_J/Pred_W)/(Obs_J/Obs_W), respectively.

Supplemental 13D. Predicted and Observed PK Profiles of Drug H In Westerner, Chinese, and Japanese

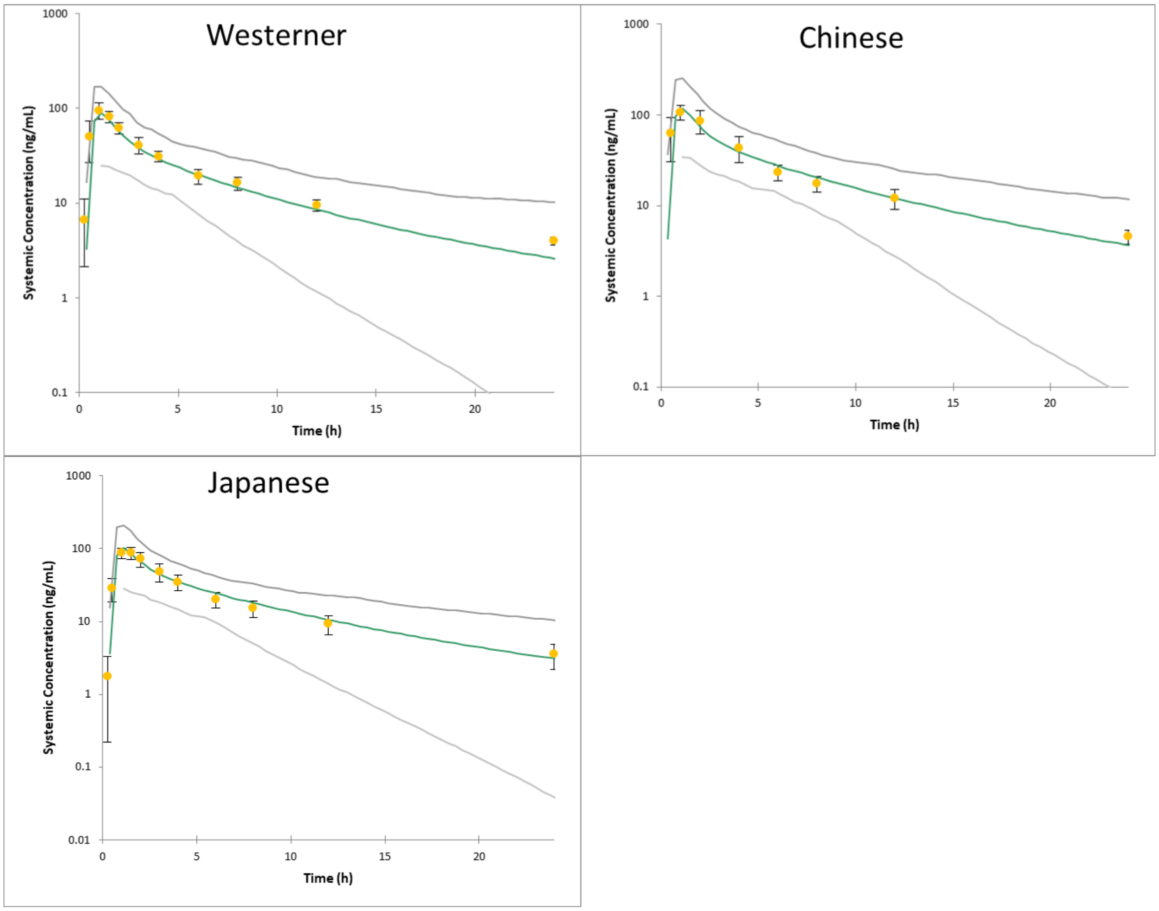

Green lines represent the predicted mean; gray lines represent the predicted 5th and 95th percentile; orange circle and error bar represent the observed mean and standard deviation.

Supplemental 14A. Drug I Input Parameters for SimCYP Simulation

**Parameters**	**Value**
Molecular weight (g/mol)	341.5
LogP	3.7
Compound type	Monoprotic base
pK_a	10.5
Fu_{plasma}	0.49
B/P ratio	1

Fa	0.7
k_a (h^{-1})	0.17
T_{lag} (h)	0
Fu_{gut}	1
Q_{gut} (L/h)	9.75
V_{ss} (L/kg)	2.4
CL_{int} (CYP2D6), $\mu L \cdot min^{-1} \cdot pmol^{-1}$	2.067
CL_{int} (CYP3A4), $\mu L \cdot min^{-1} \cdot pmol^{-1}$	0.121
CL_r (L/h)	14.4

Supplemental 14B. Predicted and Observed PK Parameters of Drug I In Westerner, Chinese, and Japanese

**Population/Dose**		**C_{max} (ng/mL)**	**AUC_{inf} (ng*h/mL)**	**$t_{1/2}$ (h)**	**C_{trough} (ng/mL)**
**Westerner**	**Observed**	**4.69**	**52.6**	**7.21**	**NA**
**(8 mg PO)**	**Predicted**	**4.37**	**59.59**	**4.68**	**NA**
**Japanese**	**Observed**	**10.9**	**113**	**7.56**	**NA**
**(16 mg PO)**	**Predicted**	**11.5**	**134**	**4.64**	**NA**

C_{max} and AUC_{inf} are reported as geometric mean; $t_{1/2}$ is reported as arithmetic mean

Supplemental 14C. Ratios of Predicted and Observed PK Parameters of Drug I In Japanese vs Westerner

	**Japanese vs Westerner**		
	**Pred_J/Pred_W**	**Obs_J/Obs_W**	**Ratio**
**C_{max}**	**1.32**	**1.16**	**1.14**
**AUC_{inf}**	**1.12**	**1.07**	**1.05**

**$t_{1/2}$**	**0.99**	**1.05**	**0.94**
**C_{trough}**	**NA**	**NA**	**NA**

Japanese dose normalized to 8 mg.

Obs_J and Obs_W are observed in Japanese and Westerner, respectively; Pred_J and Pred_W are predicted in Japanese and Westerner, respectively; Ratio is (Pred_J/Pred_W)/(Obs_J/Obs_W).

Supplemental 14D. Predicted and Observed PK Profiles of Drug I In Westerner, Chinese, and Japanese

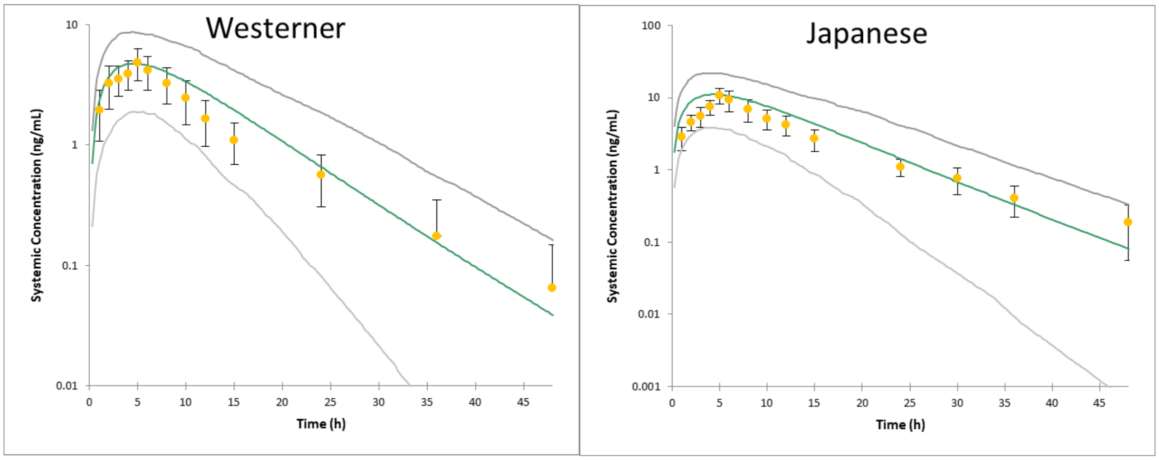

Green lines represent the predicted mean; gray lines represent the predicted 5th and 95th percentile; orange circle and error bar represent the observed mean and standard deviation.

Supplemental 15A. Drug J Input Parameters for SimCYP Simulation

**Parameters**	**Value**
Molecular weight (g/mol)	474.58
LogP	2.97

Compound type	Monoprotic base
pK_a	6.5
Fu_{plasma}	0.036
B/P ratio	0.63
Fa	1
k_a (h^{-1})	2.58
T_{lag} (h)	0
Fu_{gut}	0.383
Q_{gut} (L/h)	10.17
V_{ss} (L/kg)	1.2
CL_{int} (CYP2C9), $\mu L \cdot min^{-1} \cdot pmol^{-1}$	1.511
CL_{int} (CYP3A4), $\mu L \cdot min^{-1} \cdot pmol^{-1}$	2.864
CL_r (L/h)	0.72

Supplemental 15B. Predicted and Observed PK Parameters of Drug J In Westerner, Chinese, and Japanese

**Population/Dose**		**C_{max} (ng/mL)**	**AUC_{inf} (ng*h/mL)**	**$t_{1/2}$ (h)**	**C_{trough} (ng/mL)**
**Westerner**	**Observed**	**75.74**	**184**	**2.51**	**NA**
**(20 mg PO)**	**Predicted**	**67.81**	**211.1**	**1.79**	**NA**
**Chinese**	**Observed**	**296**	**765**	**2.82**	**NA**
**(50 mg PO)**	**Predicted**	**195**	**764**	**2.09**	**NA**
**Japanese**	**Observed**	**166**	**450**	**2.33**	**NA**
**(50 mg PO)**	**Predicted**	**225**	**907**	**2.32**	**NA**

C_{max} and AUC_{inf} are reported as geometric mean; $t_{1/2}$ is reported as arithmetic mean

Supplemental 15C. Ratios of Predicted and Observed PK Parameters of Drug J In Chinese and Japanese vs Westerner

**Chinese vs Westerner**	**Japanese vs Westerner**

	Pred_C/Pred_W	Obs_C/Obs_W	Ratio	Pred_J/Pred_W	Obs_J/Obs_W	Ratio
C_{max}	1.15	1.56	0.74	1.33	0.88	1.51
AUC_{inf}	1.45	1.66	0.87	1.72	0.98	1.76
$t_{1/2}$	1.17	1.12	1.04	1.30	0.93	1.40
C_{trough}	NA	NA	NA	NA	NA	NA

Westerner dose normalized to 50 mg for calculation of ratios.

Obs_C, Obs_J and Obs_W are observed in Chinese, Japanese, and Westerner, respectively; Pred_C, Pred_J, and Pred_W are predicted in Chinese, Japanese, and Westerner, respectively; Ratio is (Pred_C/Pred_W)/(Obs_C/Obs_W) and (Pred_J/Pred_W)/(Obs_J/Obs_W), respectively.

Supplemental 15D. Predicted and Observed PK Profiles of Drug J In Westerner, Chinese, and Japanese

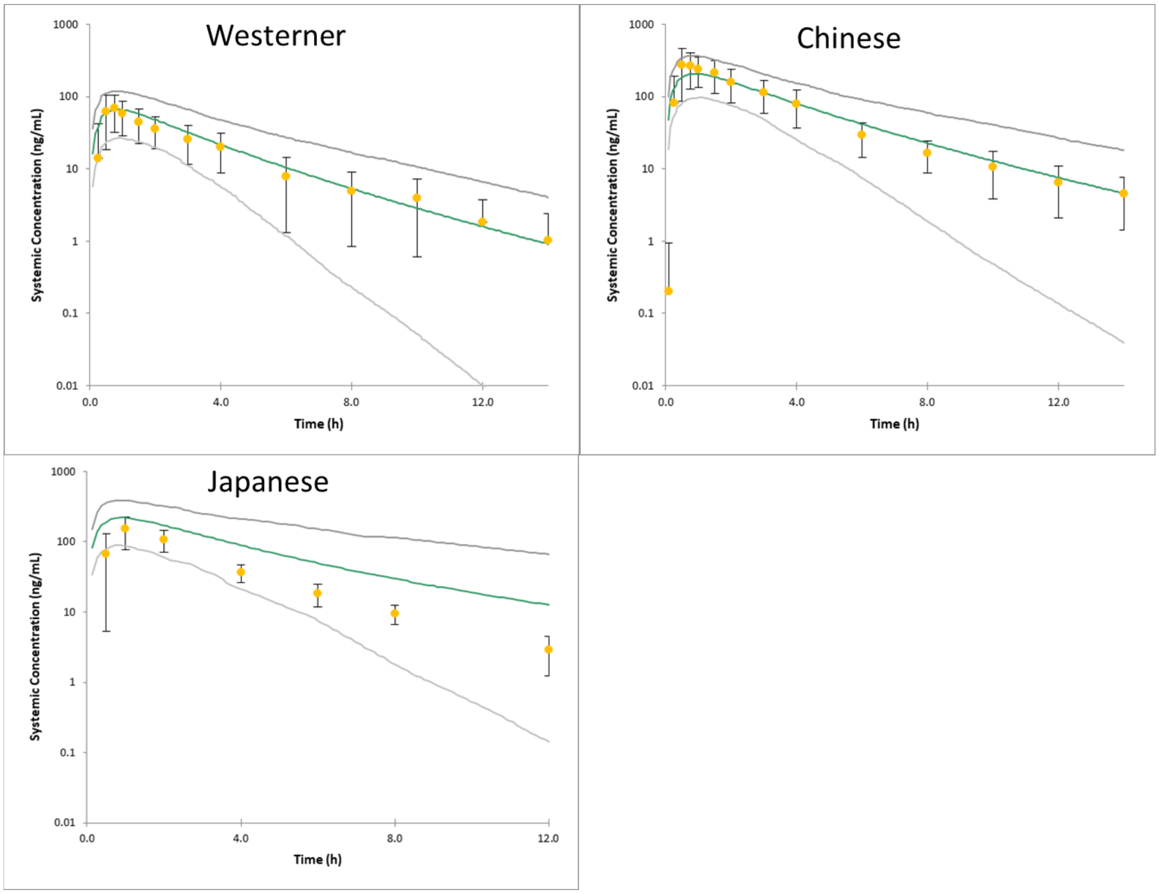

Green lines represent the predicted mean; gray lines represent the predicted 5th and 95th percentile; orange circle and error bar represent the observed mean and standard deviation.

Supplemental 16A. Drug K Input Parameters for SimCYP Simulation

Parameters	Value
Molecular weight (g/mol)	211
LogP	1.1
Compound type	Monoprotic base
pK_a	9.9
Fu_{plasma}	0.8

B/P ratio	1
Fa	1
k_a (h^{-1})	1.2
T_{lag} (h)	0
Fu_{gut}	1
Q_{gut} (L/h)	14.96
V_{ss} (L/kg)	2
$CL_{int,Hep}$, μL·min^{-1}·million^{-1}	0.3394
CL_r (L/h)	7

Supplemental 16B. Predicted and Observed PK Parameters of Drug K In Westerner, Chinese, and Japanese

**Population/Dose**		**C_{max} (ng/mL)**	**AUC_{inf} (ng*h/mL)**	**$t_{1/2}$ (h)**	**C_{trough} (ng/mL)**
**Westerner**	**Observed**	**4.48**	**97.6**	**20.0**	**NA**
**(1 mg PO)**	**Predicted**	**5.35**	**89.17**	**10.11**	**NA**
**Chinese**	**Observed**	**4.9**	**89.1**	**15.23**	**4.58**
**(1 mg PO)**	**Predicted**	**6.46**	**101**	**9.42**	**5.34**
**Japanese**	**Observed**	**5.3**	**42.5[a]**	**NA**	**7.261**
**(1 mg PO)**	**Predicted**	**5.98**	**53.7[a]**	**NA**	**5.04**

a: AUC_{0-12}

C_{max}, C_{trough}, and AUC are reported as geometric mean; $t_{1/2}$ is reported as arithmetic mean; steady state C_{trough} is derived from multiple dose of Drug K at 1 mg PO twice daily doses in Chinese and Japanese.

Supplemental 16C. Ratios of Predicted and Observed PK Parameters of Drug K In Chinese and Japanese vs Westerner

	Chinese vs Westerner			Japanese vs Westerner		
	Pred_C/Pred_W	Obs_C/Obs_W	Ratio	Pred_J/Pred_W	Obs_J/Obs_W	Ratio
C_{max}	1.21	1.09	1.11	1.12	1.18	0.95
AUC_{inf}	1.13	0.91	1.24	NA	NA	NA
$t_{1/2}$	0.93	0.76	1.22	NA	NA	NA
C_{trough}	NA	NA	NA	NA	NA	NA

Obs_C, Obs_J and Obs_W are observed in Chinese, Japanese, and Westerner, respectively; Pred_C, Pred_J, and Pred_W are predicted in Chinese, Japanese, and Westerner, respectively; Ratio is (Pred_C/Pred_W)/(Obs_C/Obs_W) and (Pred_J/Pred_W)/(Obs_J/Obs_W), respectively.

Supplemental 16D. Predicted and Observed PK Profiles of Drug K In Westerner, Chinese, and Japanese

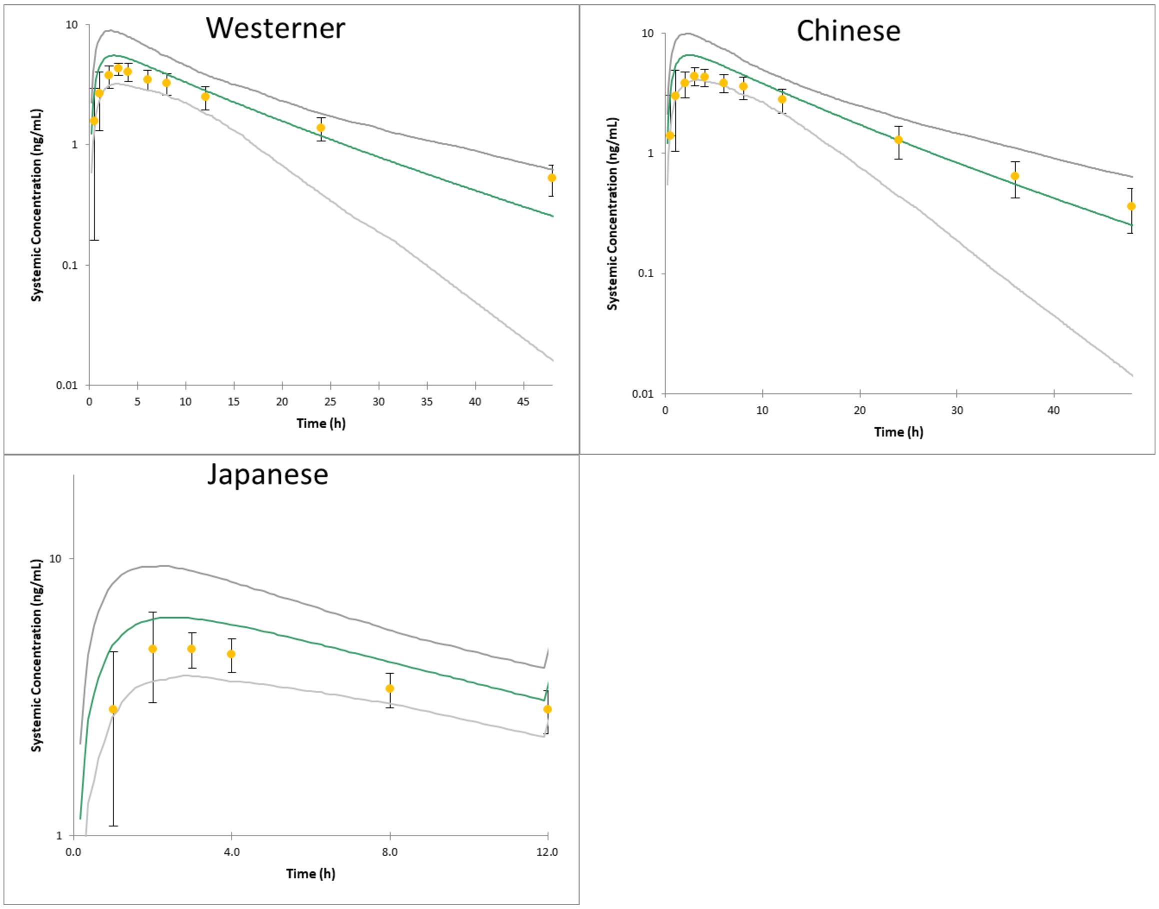

Green lines represent the predicted mean; gray lines represent the predicted 5th and 95th percentile; orange circle and error bar represent the observed mean and standard deviation.

Supplemental 16E. Predicted and Observed Ctrough of Drug K In Chinese, and Japanese

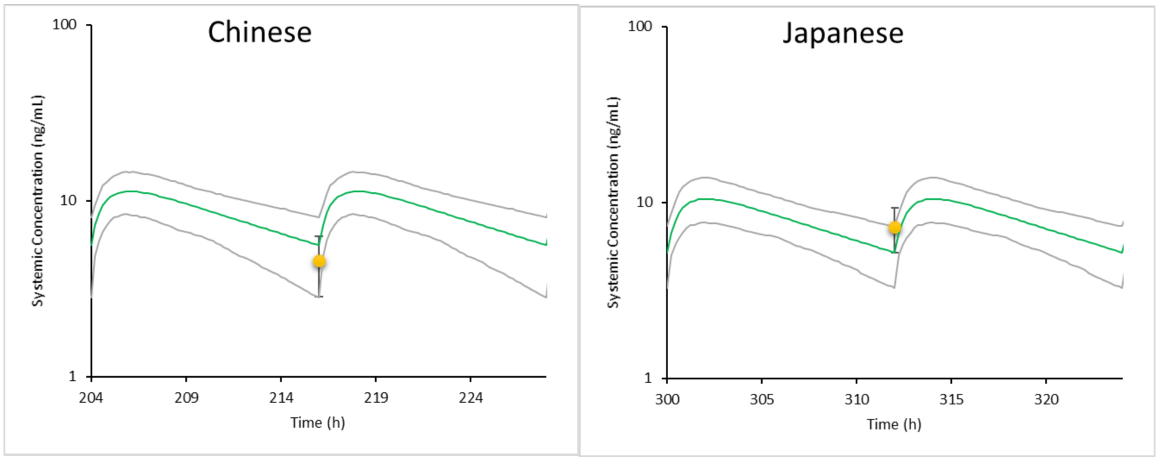

Green lines represent the predicted mean; gray lines represent the predicted 5th and 95th percentile; orange circle and error bar represent the observed mean and standard deviation.

Supplemental 17A. Drug M Input Parameters for SimCYP Simulation

**Parameters**	**Value**
Molecular weight (g/mol)	349
LogP	1.8
Compound type	Monoprotic base
pK_a	1.63
Fu_{plasma}	0.42
B/P ratio	0.89
Fa	0.96
k_a (h^{-1})	1.44
T_{lag} (h)	0
Fu_{gut}	1
Q_{gut} (L/h)	14.61
V_{ss} (L/kg)	1.88

V_{max} (CYP2C9), $\mu L \cdot min^{-1} \cdot pmol^{-1}$	0.1
K_m (CYP2C9), μM	11
V_{max} (CYP2C19), $\mu L \cdot min^{-1} \cdot pmol^{-1}$	7
K_m (CYP2C19), μM	3.5
V_{max} (CYP3A4), $\mu L \cdot min^{-1} \cdot pmol^{-1}$	0.31
K_m (CYP3A4), μM	15

Supplemental 17B. Predicted and Observed PK Parameters of Drug M In Westerner, and Japanese Separated by CYP2C19 Polymorphisms

Population/Dose		C_{max} (µg/mL)	AUC_{0-12} (µg*h/mL)	$t_{1/2}$ (h)	C_{trough} (µg/mL)
Westerner - EM	Observed	2.22	10.8	NA	0.36
(200 mg PO)	Predicted	1.57	13.4	NA	0.62
Westerner - HEM	Observed	2.82	17.6	NA	0.75
(200 mg PO)	Predicted	2.76	26.5	NA	1.5
Westerner - PM	Observed	6.84	61.6	NA	4.27
(200 mg PO)	Predicted	4.12	43.4	NA	2.97
Japanese - EM	Observed	2.15	12	NA	0.49
(200 mg PO)	Predicted	1.87	16.2	NA	0.77
Japanese - HEM	Observed	3.36	20	NA	0.97
(200 mg PO)	Predicted	3.18	31	NA	1.81
Japanese - PM	Observed	6.87	65	NA	4.74
(200 mg PO)	Predicted	5.94	65.2	NA	4.69

C_{max} and AUC_{0-12} are reported as geometric mean; $t_{1/2}$ is reported as arithmetic mean; steady state C_{trough} is derived from multiple dose of Drug M at 200 mg PO twice daily doses in Westerner and Japanese.

Supplemental 17C. Ratios of Predicted and Observed PK Parameters of Drug M In Japanese vs

Westerner Separated by CYP2C19 Polymorphisms

	Japanese vs Westerner - EM			Japanese vs Westerner - HEM			Japanese vs Westerner - PM		
	Pred_J/ Pred_W	Obs_J/ Obs_W	Ratio	Pred_J/ Pred_W	Obs_J/ Obs_W	Ratio	Pred_J/ Pred_W	Obs_J/ Obs_W	Ratio
C_{max}	1.19	0.97	1.23	1.15	1.19	0.97	1.44	1.00	1.44
AUC_{0-12}	1.21	1.11	1.09	1.17	1.14	1.03	1.50	1.06	1.42
$t_{1/2}$	NA	NA	NA	NA	NA	NA	NA	NA	NA
C_{trough}	1.24	1.36	0.91	1.21	1.29	0.94	1.58	1.11	1.42

Obs_J and Obs_W are observed in Japanese and Westerner, respectively; Pred_J and Pred_W are predicted in Japanese and Westerner, respectively; Ratio is (Pred_J/Pred_W)/(Obs_J/Obs_W).

Supplemental 17D. Predicted and Observed PK Profiles and Ctrough of Drug M In Westerner, and Japanese Separated by CYP2C19 Polymorphisms

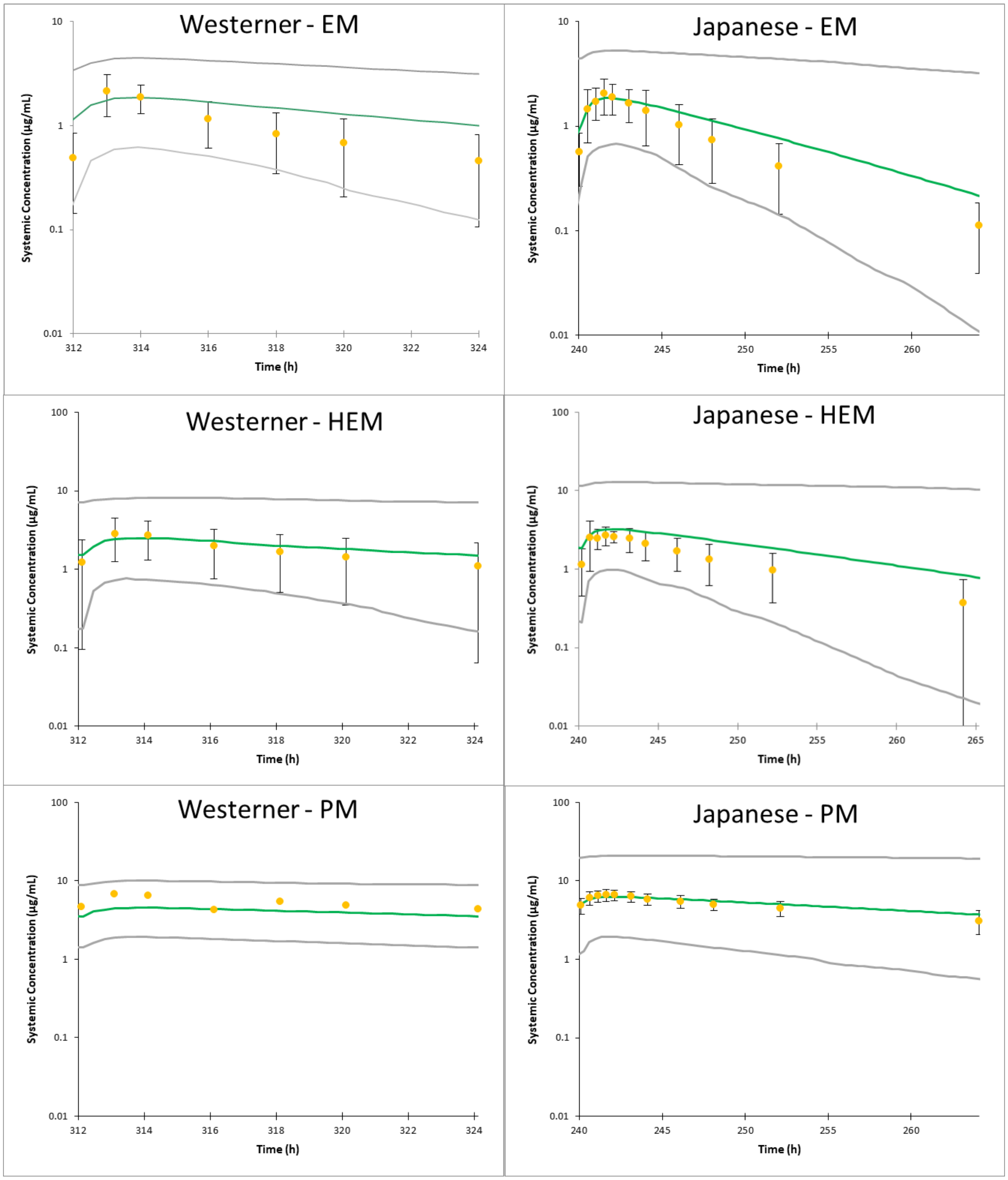

Green lines represent the predicted mean; gray lines represent the predicted 5th and 95th percentile; orange circle and error bar represent the observed mean and standard deviation.
